# Characterising support and care assistants in formal hospital settings: a scoping review

**DOI:** 10.1186/s12960-023-00877-7

**Published:** 2023-11-27

**Authors:** Vincent A. Kagonya, Onesmus O. Onyango, Michuki Maina, David Gathara, Mike English, Abdulazeez Imam

**Affiliations:** 1 Health Services Unit, KEMRI-Wellcome Trust Research Programme, Nairobi, Kenya; 2 Nuffield Department of Medicine, Health Systems Collaborative, University of Oxford, Oxford, United Kingdom; 3 MARCH Centre, London School of Hygiene and Tropical Medicine, London, United Kingdom

**Keywords:** Care assistant, Human resources for health, Task-shifting, Health workforce

## Abstract

**Background:**

A 15 million health workforce shortage is still experienced globally leading to a sub-optimal healthcare worker-to-population ratio in most countries. The use of low-skilled care assistants has been suggested as a cost-saving human resource for health strategy that can significantly reduce the risks of rationed, delayed, or missed care. However, the characterisation, role assignment, regulation, and clinical governance mechanisms for unlicensed assistive workforce remain unclear or inconsistent. The purpose of this study was to map and collate evidence of how care assistants are labelled, utilised, regulated, and managed in formal hospital settings as well as their impact on patient care.

**Methods:**

We conducted a scoping review of literature from PUBMED, CINAHL, PsychINFO, EMBASE, Web of Science, Scopus, and Google Scholar. Searches and eligibility screening were conducted using the Participants–Context–Concepts framework. Thematic content analysis guided the synthesis of the findings.

**Results:**

73 records from a total of 15 countries were included in the final full-text review and synthesis. A majority (78%) of these sources were from high-income countries. Many titles are used to describe care assistants, and these vary within and across countries. On ascribed roles, care assistants perform direct patient care, housekeeping, clerical and documentation, portering, patient flow management, ordering of laboratory tests, emergency response and first aid duties. Additional extended roles that require higher competency levels exist in the United States, Australia, and Canada. There is a mixture of both positive and negative sentiments on their impact on patient care or nurses’ perception and experiences. Clinical and organisational governance mechanisms vary substantially across the 15 countries. Licensure, regulatory mechanisms, and task-shifting policies are largely absent or not reported in these countries.

**Conclusions:**

The nomenclature used to describe care assistants and the tasks they perform vary substantially within countries and across healthcare systems. There is, therefore, a need to review and update the international and national classification of occupations for clarity and more meaningful nomenclature for care assistants. In addition, the association between care assistants and care outcomes or nurses’ experience remains unclear. Furthermore, there is a dearth of empirical evidence on this topic from low- and middle-income countries.

**Supplementary Information:**

The online version contains supplementary material available at 10.1186/s12960-023-00877-7.

## Introduction

### Background information

Service delivery in hospitals is labour-intensive and human resource requirements represent a disproportionate allocation of any availed health service budget [1, 2]. Whereas achieving optimal healthcare worker-to-patient ratios remains a global challenge [3–5], the majority of health systems are recognising the importance of a cost-effective and safe health workforce. However, inadequate finances for human resource recruitment and development [6, 7], limited capacity development opportunities, poor remuneration, difficult working conditions, and limited career advancement opportunities [7, 8] all contribute to the inability to attain optimal staffing ratios in healthcare facilities.

Broadly, the concept of human resources for health (HRH) not only includes primary care providers (i.e., physicians, nurses, or pharmacists) but also other assistive personnel like the administrators and care assistants who may not directly provide care to patients but provide operational support services and are, therefore, crucial to service delivery and the overall functioning of the healthcare system [8]. In 2020, the World Health Assembly [9] acknowledged that concerted efforts, including the implementation of the WHO Global Strategy on HRH [8] led to a reduction of shortages in healthcare workers by 3 million to 15 million [10]. Unlike other regions in the global north, countries east of the Mediterranean Sea and sub-Sahara Africa still experience dire staffing shortages and comparatively low healthcare worker-to-population ratios [7, 9].

The use of assistive personnel has been suggested as a task-shifting and skill-mix initiative that can help professional healthcare workers to optimise their shift time to focus on high-acuity or more technical tasks [11–16]. Conceptually, task-shifting is the rational re‐distribution or delegation of specific tasks among health workforce teams from the highly skilled to the less qualified/skilled staff [13]. On the other hand, skill-mix has been described as a multi-dimensional undertaking that incorporates performative elements (such as knowledge, skills, abilities, and competencies), intra-professional transversality (such as grade, level of expertise, education, and training), and inter-professional transversality of healthcare practice(i.e., a mix of posts, regulation, staff mix, and ratios). The relative proportions of highly skilled care providers and less skilled support staff represent an example of a staffing skill-mix. The current evidence suggests skill-mix tends to vary by fiscal year and country, and this is partly attributed to the adequacy of healthcare financing for HRH [7, 8, 17]. Consequently, to manage scarce human and financial resources efficiently while delivering needed care, healthcare systems adopt task-shifting and skill-mix strategies that target lower-level cadres whose emoluments might be less costly.

Some evidence suggests the nature and roles ascribed to unlicensed assistive staff vary across countries and healthcare systems. For example, whereas some hospitals in high-income countries assign their care assistants extended roles, such as phlebotomy and patient monitoring [18–20], in low-income settings, they seem to take up informally negotiated basic duties, such as general housekeeping, portering [18, 21–23], or supporting patients to perform activities of daily living [11]. Often, these assistants do not undergo any formal or professional pre-service training [8, 13, 24] based on a standard curriculum but might have different forms of informal or on-the-job training, mainly from registered nurses. However, a lot remains unclear or inconsistent on their scope of duties.

Some existing literature has examined the utilization of care assistants and highlighted knowledge gaps on how they are deployed in hospitals and how they affect patient care outcomes and experiences [25]. In addition, available reviews [21, 26, 27] have focused more on unlicensed assistive personnel who have undergone some form of structured formal training ranging from 6 months up to 2 years [11]. No review has focused on those with no formal pre-service training [8, 13, 24], which are a common cadre in many under-resourced healthcare settings, where clinical governance, licensure, and frameworks to regulate their activities may be limited [26]. Moreover, despite the wide utilisation of care assistants in hospitals, the global strategy on HRH hardly mentions the roles care assistants perform [11]. This leaves a lacuna in the clarity of their roles, while the scope of practice is mostly at the discretion of individual hospitals or supervisor. The use of this cadre of staffers is not invariable across most hospitals and begs for scrutiny. Thus, in this review, we coined and used the term hospital-based “ward/care assistants” (CAs) to describe this cadre of staff—lower-skilled assistive personnel who typically provide support to nurses in formal hospital settings. Consequently, this scoping review sought to answer the following questions:

### Review questions

In formal hospital settings:

1. What roles and duties are performed by ward/care assistants?
2. What impact do ward/care assistants have on patient care?
3. What are the perceptions and experiences of nurses towards ward/care assistants?
4. What clinical or organisational governance frameworks exist to regulate the activities of ward/care assistants?

## Methods

### Design

We conducted a scoping review following the Joanna Briggs Institute and PRISMA–ScR evidence synthesis and reporting guidelines [28–30]. These guidelines describe the best practices for evidence synthesis from protocol development, search strategies, data extraction, interpretation, and reporting of scoping review results.

### Protocol registration

The protocol for this review was registered on the Open Science Framework registries [31].

#### Search strategy and data sources

We conducted our literature search in PUBMED, CINAHL, PsychINFO, EMBASE, Web of Science, and Scopus with additional targeted searches from Google Scholar and citation chasing, particularly for grey literature. The last search was conducted on 20^th^ June 2022. We combined the search terms using Boolean operators and adapted them for each of the electronic databases. The comprehensive list of the keywords used is shared in Additional File 1 and a sample full search strategy in Additional File 2. There was no time limitation on the search period. However, language was restricted to English-published papers only. If a full text was completely irretrievable, the reviewers attempted to contact the corresponding author for the full text, otherwise, the paper was excluded from charting and synthesis.

#### Eligibility criteria and data items

We used the Participants–Concept–Context framework [32, 33] to describe the inclusion and exclusion criteria. Under “Participants”, we only included papers that reported on CAs. We defined CAs as hospital-based staff that support healthcare professionals to provide non-clinical, low-skilled basic tasks to a patient or within a ward or clinic—but mainly supporting nurses’ work. They would ordinarily not have any formal professional, technical training before working, licensing, or regulatory requirements save for a high-school level education with some level of on-the-job (in-service) training, particularly from nurses. We, therefore, excluded literature that reported on CAs who had undergone some formal or professional pre-service training [8, 13, 24] or who require licensure before working. Pre-service training was considered formal or professional if there is a defined curriculum and the training duration exceeded 6 months.

Under “Concepts”, we sought to document the characterisation of CAs as unlicensed assistive personnel in formal hospital settings, their ascribed roles and duties, and their impact on patient care and nurses’ experience. We also sought to map regulatory frameworks and clinical governance mechanisms at the workplace. We only included papers that reported on these review objectives.

Finally, for “Context” we considered studies that report CAs working or providing support to nurses in both in-patient and outpatient care settings, including nursing care homes. CAs providing services in the community or individuals’ homes of their patients—also known as community home-based care—were excluded. In addition, literature from across the globe irrespective of the World Bank’s income group [34] was included. We considered both primary and secondary research papers as well as relevant grey literature. Thesis and dissertations, conference abstracts, seminar reports, case reports/series, and animal studies were excluded from this review. Additional File 3 provides a detailed description of the eligibility criteria.

#### Data management and synthesis

Data management and analysis steps involved study selection, data extraction, data synthesis, and reporting.

#### Study screening and selection

**Fig. 1:**
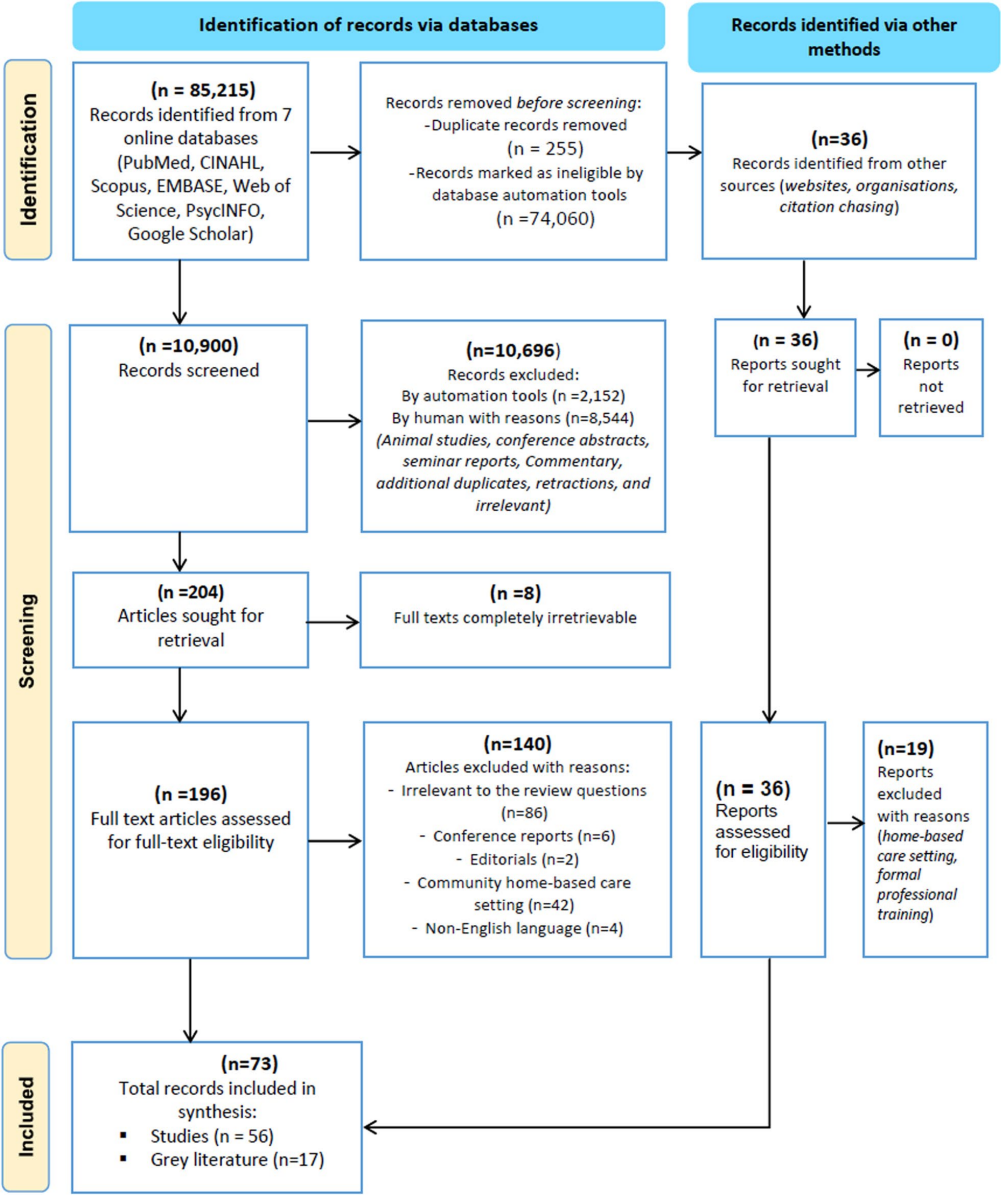
Summary of search results and records screening

Two reviewers (VK and OO) independently conducted literature searches in June 2022. The search results were then consolidated and screened for relevance against the eligibility criteria, initially by title and abstract then later by full text. The screening stage was done independently by two reviewers (VK and OO) with the aid of The EndNote reference manager [35]. Reasons for ineligibility were documented at the full-text screening stage and are reported in the results section (Fig. 1). Inconsistencies or disagreements on the eligibility of a paper were jointly discussed between the two reviewers at both the abstract and full-text review stages. In case inconsistencies or disagreements could not be resolved by the two reviewers, an additional third reviewer (AI) was invited to act as a tiebreaker in a joint discussion for consensus building.

#### Charting the data

Two reviewers (VK and OO) used a pre-developed template on Microsoft Excel to aid in the charting of key pre-specified study information crucial in answering the review questions, including bibliographic details, study context and characteristics, concepts, roles and duties, training, regulatory/governance mechanisms, patient care outcomes, nurses’ experiences related to the utilisation of the CAs. Charting was done jointly by two reviewers (VK and OO). The synthesis, interpretation, and reporting of the findings of this review were then guided by the PRISMA–ScR guidelines [29].

### Critical appraisal of the individual papers

Quality and risk of bias assessment were not undertaken, since our eligible papers yielded multiple types of papers and methods that were heterogenous in methodology, region, care setting, participant selection, and reporting of their findings (illustrated in Additional File 4). This would have meant multiple critical appraisal tools. Moreover, quality assessment is not a mandatory requirement for scoping reviews, since they do not aim to synthesise the ‘strength’ of evidence from literature but rather to provide an overview of the available evidence[36]. Thus, the reviewers agreed to include all the 73 eligible papers in data charting and synthesis.

### Synthesis

The Joanna Briggs Institute’s evidence synthesis manual [28, 30] guided our synthesis approach. With the aid of NVIVO 12 Plus [37], we conducted a thematic content analysis. Data from the charting template was uploaded onto the NVIVO 12 Plus program followed by open coding onto nodes that helped to answer our review questions. The nodes were then refined and grouped into sub-themes and themes. The process generated themes on how CAs are characterised, their ascribed duties, patient care outcomes, and sentiments (i.e., views, feelings, or opinions) of nurses’ regarding their experiences working with CAs. The emerging themes were then assigned sentiment labels on whether they had a positive, neutral, or negative effect on care or experiences of care. Frequencies of mention of titles that characterise CAs were generated from each source and presented in a word cloud. Similarly, each specific task charted from individual sources was coded into a theme on tasks. These specific tasks were then grouped into broad categories for easier interpretation and reporting. The number of records mentioning a task and the task frequency across the records were then generated for graphical presentation. Finally, clinical, and organisational governance mechanisms were extracted, curated, and tabulated as reported in the individual sources.

## Results

Reporting is guided by the Preferred Reporting Items for Systematic Reviews and Meta-Analysis extension for Scoping Reviews (PRISMA–ScR) guidelines [29].

### Search and screening results

Figure 1 shows a flow diagram adapted from the PRISMA 2020 statement [38]*.* It provides a summary of the sources searched and the records assessed for eligibility. We obtained a total of 85,251 records from electronic databases and grey literature searches. After screening for eligibility and excluding records based on the eligibility criteria, a total of 73 records were included in the final full-text review, data charting, and synthesis.

### Summary statistics

Table 1 provides a summary of key characteristics of the included records.

**Table 1:** Characteristics of included records

	Number of papers *n*
Publication periods	*n* (%)
After 2020	9 (12)
2016–2020	**18 (24)**
2011–2015	**22 (29)**
2006–2010	9 (12)
2000–2005	9 (12)
Before 2000	6 (8)
Type of papers/methods	*n* (%)
Observational* (prospective, retrospective, cross-sectional, surveys)*	**27 (37)**
Grey literature*	**18 (25)**
Qualitative	9 (12)
Reviews (Systematic, Scoping, Integrative)	8 (11)
Mixed Methods	4 (5)
Quasi-experimental	3 (4)
Case study	3 (4)
Ecological study	1 (1)
Source of papers (World Bank’s income group classifications)	*n* (%)
High-income countries	**57 (78)**
Upper middle-income countries	1 (1)
Lower middle-income countries	4 (5)
Low-income countries	2 (3)
Global**	9 (12)

^*^Includes white papers, position papers, policy brief, training guide

^**^Not specified to any income region or group; These are mostly reviews and white papers from WHO which mainly have a global focus. In bold is the majority proportion of the sources

### Characteristics of sources

Majority of the papers were observational studies followed by grey literature. Records from high-income settings accounted for a majority of the eligible full texts. That is, United Kingdom (UK) had the highest number of sources (*n* = 19 [25%]) followed by the United States of America (*n* = 13 [17%]) and Australia (*n* = 7 [9%]), respectively. Japan, Israel, Benin, Hong Kong, Malawi, Brazil, and Uganda had 1 report each.

### The hospital setting

Only 47 (n = 64%) of the papers described the type of care setting. Our synthesis established that these relevant sources yielded a mix of rural and urban (*n* = 22), inpatient-only (n = 18) and outpatient-only (*n* = 2), nursing care homes (*n* = 12), both inpatient and outpatient (*n* = 9) with a mix of children and adult care settings. Moreover, of the sources that mentioned a type of setting, public and private hospitals were reported in 30 (40%) and 13 (17%) papers, respectively. Twelve papers (16%) had a mix of both private and public. Additional File 4 provides more information on the setting as reported in the included papers.

### Characterising CAs

**Fig. 2:**
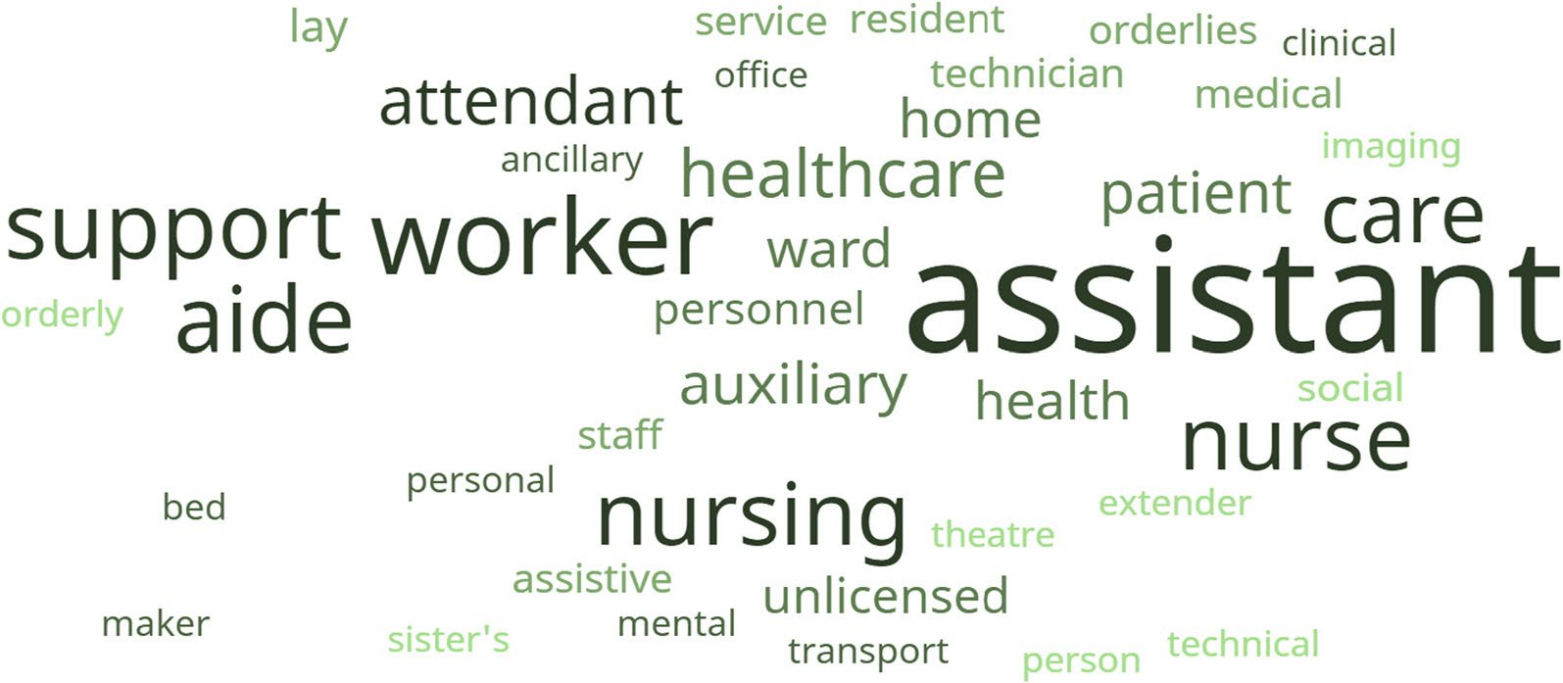
Common descriptors for CAs

Conceptually, we note that many titles are currently used for the identification or description of CAs in different countries and regions (Additional File 5). Only a few select similarities exist, for instance, “nursing assistant” is used in Australia, Hong Kong, China, New Zealand, the United States of America (USA), and the UK. UK has the highest number of terms/name variations that describe CAs (> 21), followed by Australia and Canada (> 11 each) then the USA and Hong Kong China (> 7 each). Consequently, the term “Assistant” is the most common stem word followed by “support staff/worker” in what are typically compound terms for CAs. Figure 2 shows a word cloud illustration of these variations. The other variations are summarised in Additional File 6 and in order of the most common to the least common.

### Objective 1: Duties performed to CAs in hospital settings

#### Duties ascribed to CAs

A majority (78%, *n* = 58) of the records mentioned specific tasks performed by CAs in their care setting or facility. Our review was able to chart and curate 58 different tasks and these were grouped into 7 broad categories (illustrated in Table 2). That is, direct patient care (*n* = 53 records), housekeeping (*n* = 26 records), clerical and portering (*n* = 19 records each), patient flow management and ordering laboratory tests (*n* = 4 records each). Emergency response and first aid was the least reported task category.

**Table 2:** Duties undertaken by CAs

Broad category	Sub-category	Specific tasks
Direct Patient Care^*(Reported in 53 records)*^	• Vital signs monitoring^#^	• Vital signs taking (unspecified)^#^ • Blood pressure measurement^#^ • Blood glucose check^#^ • ECG monitoring^#^ • Temperature check* • Pulse rate and oxygen saturation(SP02) * • Fluid balance*
	• Support patient with hygiene^#^ (bathing, dressing, oral care) • Feeding (oral) and utensils cleaning^#^ • Patient assessment and behavioural observations* • Communication and health promotion messaging*	**NA**
	• Specimen/sample collection	• Peripheral venepuncture^#^ (phlebotomy) • Unspecified sample collection*
	• Elimination needs support	• Bed pans and urinal emptying^#^ • Continence care* • Colostomy care*
	• Anthropometric measurements	• Weight check^#^ • Height check^#^ • Waist circumference measurements* • Body mass index measurements*
	• Medication (drug administration)	• Intramuscular injections* • Flu vaccination* • Medication* (unspecified)
	• Physical exercise (ambulation) and physiotherapy^#^ • Bed making and patient turning^#^ • Wound care and cord cleaning^#^ • Psychosocial and emotional support^#^ • Serving meals^#^ • Catheterization • NGT or OGT feeding • Eye care • Supporting with admissions process • Supporting with discharge process • Resuscitation and basic life support or first aid* • Last office care* • Prepare patients for procedures*	NA
Housekeeping^#^ ^*(Reported in 26 records)*^	• Cleaning environment and surfaces^#^ • Stock taking • Equipment checks and cleaning • Setting up of rooms for procedures • Linen management • Medical waste disposal • Care of patients' properties and valuables* • Properties and valuables custody*	NA
Clerical and Documentation ^*(Reported in 19 records)*^	• Documentation • Telephone calls	NA
Portering ^*(Reported in 19 records)*^	• Transportation	NA
Patient Flow Management ^*(Reported in 4 records)*^	• Patient flow management^#^ • Bookings and scheduling*	NA
Order Laboratory Tests* ^*(Reported in 4 records)*^	• Unspecified laboratory tests • Urinalysis*	NA
Emergency Response and support* ^*(Reported in 2 records)*^	NA	NA

*NA* Not Applicable

^#^Most commonly reported task

^*^Least commonly reported task

Table 2 unpacks the specific tasks performed under broad and sub-categories. It was noted that vital signs monitoring, patient hygiene, and feeding are top of the list of direct care duties, whereas environment and surface cleaning, stock taking, equipment care and device functionality checks are the most common housekeeping tasks. However, a few outstanding papers had CAs performing more extended roles that require an extra level of knowledge and skills, i.e., flu vaccination, drug injections [24, 39, 40], catheterisation [24, 40–44], phlebotomy [44–49], electrocardiogram (ECG) monitoring [39, 44, 46, 49–52], wound/colostomy care [24, 27, 39–42, 44, 49, 51, 53], resuscitation [54], and requesting laboratory tests [52, 55–57]. These extended roles are observed mainly in high-income countries (Australia, Canada, and UK, and the USA) and not in low- and middle-income countries (LMICs).

### Objective 2a: Impact of CAs on patient care and their experiences

Only 20 (27%) papers reported some form of patient care outcomes. With the aid of NVIVO 12 Plus [37], a thematic analysis approach was used to examine and curate the impact of CAs on patient care. The emerging themes were then assigned sentiment labels on whether they had a positive, neutral, or negative effects on care. As shown in Table 3, the papers report a mix of both positive and negative effects.

**Table 3:** Impact of CAs on patient care

	Theme	Impact/effect	Sentiment
1	Timeliness and Efficiency of Care	-Tasks undertaken and completed on time [46]	Positive
		-Reduced waiting times [52, 58]	
		-Shorter period of hospitalization [58]	
		-Longer length of hospital stay [54, 59]	Negative
2	Quality and Effectiveness of Care	-Nurses get more contact time with patients [52, 60, 61]	Positive
		-High number of services provided [58]	
		-Enhanced psychosocial and emotional care [51, 62–64]	
		-Reduced need for catheter use [65]	
		-Enhanced knowledge on care [62]	
		-Reduced in-hospital mortality [66]	
		-Reduced need for pain management [63, 65]	
		-Increased patient assessment and monitoring [55, 66]	
		-No beneficial effect [61]	Negative
		-Reduced direct nurse–patient interaction [61, 67, 68]	
3	Patient safety	-Reduced risk for complications [65, 69, 70]	Positive
		-Reduced medication errors [69]	
		-Reduced patient injury [48, 54]	
		-Risk to patient safety [42, 50, 68]	Negative
		-Medical complications (*Deep venous thrombosis, Unplanned endotracheal extubation, Falls with injuries, Increase in pressure ulcers, High rate of failure to rescue, Increase in hospital acquired infections, Increased in-hospital mortality*) [14, 51, 54, 59, 70–72]	
4	Patient Satisfaction	-Reduced anxiety [42, 50, 68]	Positive
		-Enhanced communication of health messages to patients and relatives [62, 64, 73]	
		-Increased trust and confidence [50, 52]	
		-Increased patient satisfaction [51, 55, 61]	
		-Improvement in quality of care [55, 58, 62, 63, 65, 66, 69, 74]	
		-Experience of respect and dignity [63]	
		-Misunderstandings and conflicts among patients, relatives, and staff [75]	Negative
		-Patients doubt on the competencies of ward assistant [42, 48]	
5	Cost of Care	-High hospitalization costs [59]	Negative
6	Professional identity	-Confusion of roles with nurses [48, 52]	

### Objective 2b: Nurses’ perception and experiences towards CAs

Only 23 (32%) papers reported on nurses’ experience of working with CAs. With the aid of NVIVO, a sentiment analysis approach was used to code and curate themes related to nurses’ experiences of working with CAs at the individual level. The emergent themes were grouped into either positive, neutral, or negative sentiments as illustrated in Table 4. Additional file 7 is a summary illustration of what is reported as per the synthesised records.

**Table 4:** Nurses’ sentiments on utilisation of CAs

	Theme		Sentiment
1	Effectiveness and Continuity of care	▪ Continuity of patient care [48, 54]	Positive
		▪ Few work interruptions [54]	
		▪ More tasks are completed (reduced risk for missed care) [45, 52, 59, 68, 75, 76]	
		▪ More time for high acuity nursing tasks [45, 48, 59, 75, 76]	
		▪ Reduced waiting times [48, 52]	
		▪ Covering absenteeism [51, 54, 59]	Neutral
		▪ High staff turnover among nurses (whether supplemental or substitution model) [51, 54, 59]	
		▪ Reduced nurse–patient contact time and interaction [54]	Negative
		▪ Feeling of fragmented, dehumanized care [27, 48, 77]	
2	Health workforce deployment and supervision	▪ Extra pair of hands for monitoring patient status [39]	Positive
		▪ Supplements nurse staffing [51, 54, 59]	
		▪ Replacement/Substitution for nurses [51, 54, 59]	Negative
		▪ Extra workload on supervision of delegated tasks [42, 51, 54, 59]	
		▪ More time spent on induction, training, and supervision [42, 51, 54, 59]	
		▪ Pressure to delegate due to staff shortage [76]	
		▪ Resistance, resentment, and scepticism by qualified staff [27, 48, 77]	
		▪ Nurses’ unwillingness to shift some tasks [27, 48, 77]	
		▪ Variability role assignments limits nurses' ability to effectively delegate and supervise [27, 48, 77]	
		▪ Some assistants have reading and writing difficulties [75]	
		▪ Unclear accountability lines for actions [42, 54, 78]	
3	Motivation and Job satisfaction	• Increased job satisfaction [59, 79]	Positive
		• Reduced workload via task-shifting [52, 59, 78]	
		• Nurse burnout [42, 51, 54, 59]	Negative
		• Reduced job satisfaction [54]	
4	Professional identity	• Patients confuse between professional nurse and ward assistants [48]	Negative
		• Feelings of role ambiguity(confusion) and conflict [27, 48, 77]	
		• Role deprivation and loss of professional identity [48, 52]	
5	Staff safety	• Violence and abuse towards the nurse [51, 59]	Negative
6	Inter-cadre communication	• Improved communication between nurse and assistants [64, 80]	Positive

### Objective 3: clinical and organisational governance frameworks that regulate activities of CAs

#### Objective 3a: Regulatory and clinical governance mechanisms

Only 33 (45%) of the records mentioned some form of an organisational regulatory or clinical governance mechanism for the CAs—8 in the UK [26, 27, 39, 41, 42, 48, 54, 73, 81–83], 6 in the USA [26, 51, 65, 70, 84–87], 3 were from Taiwan China [75, 88], 2 from Canada [24, 40], and 2 from Australia[26, 51], and 1 each from Japan [72], Brazil [68], Sweden [76], Kenya [89], Malawi [66], and Uganda [90]. Moreover, Brazil, Kenya, Malawi, and Uganda are the only LMICs reporting some form of clinical governance mechanism. However, for Kenya and Uganda, it is largely a proposed framework and not an already operationalised one.

We note that these mechanisms vary substantially within and across the 15 countries reviewed (Additional File 7 and Additional File 8). However, notable similarities in some countries include a requirement for completion of a competency-based training curriculum in a work setting and that there is delegation and supervision by a qualified (registered) nurse. The majority of countries lack a legislative framework that standardises or regulates the training of CAs. Moreover, nearly, all the countries (93%) do not have a task-shifting/sharing policy that guide the delegation and supervision of tasks.

### Objective 3b: Training/capacity development

Only 41 (55%) of eligible papers reported some level of training requirement (pre-service or in-service) and this was reported in several countries, including Australia, Benin, Canada, Israel, Kenya, Malawi, the Republic of Ireland, Taiwan, China, UK, and the USA. Sources from Brazil, Hong Kong, China, Sweden, Uganda, and Japan did not report any form of training requirement before or after the recruitment of CAs.

Our review establishes that nearly all the CAs are required to undertake onboarding training and continue with in-service competency skills training at their own pace. The skills development period varies substantially across all the countries reviewed. For instance, the minimum in-service (on-the-job) training duration ranged between 1 and 48 h [61, 66, 70], whereas the maximum in-service training period was undertaken between 126 and 672 days [26, 51, 91].

### Training topics/content

The majority of the theory and practical learnings covered topics and skills related to basic nursing care (e.g., taking vital signs, simple wound care, taking weight and height measurements, specimen collection, or patient hygiene), workplace health and safety (including cleanliness and basic first aid), communication skills, infection prevention and control (including equipment processing), anatomy and physiology, confidentiality, privacy, and dignity as the most common training topics. The least common topics mentioned include family support/centred care, health promotion, human growth and development, food, and nutrition, and counselling. A detailed list is found in Additional File 9.

## Discussion

This review aimed to map evidence for the characterisation of lower-skilled support and CAs in formal hospital settings. We note that there is a lot of inconsistency and substantial variation in the terms or titles for CAs, their training, the scope of practice, and regulatory mechanisms within and across the reports included from 15 countries. Moreover, we note that the two commonly used terms “assistant” and “support worker” do not align with the ILO’s ISCO 5132, 5133, and 3231 descriptions [92] that use “Institution-based personal care workers” or “nursing aid” to refer to the titles and roles ascribed to CAs. Thus, our synthesis points out the need to review the ILO’s ISCO nomenclature for this occupational group.

Our evidence mapping indicates a substantial amount of empirical literature on task-shifting/sharing between nurses and CAs in high-income countries but largely understudied in LMICs. However, overall, some evidence suggests CAs may contribute to improved quality of patient care by availing nurses time to concentrate on high-acuity and critical care activities [11, 12]. There is, however, contrary evidence suggesting the involvement of CAs in patient care may pose a risk to patient safety and quality of care [26, 48, 93, 94]. In addition, the evidence for CAs’ ascribed roles is generally mixed and their role boundary with professional nurses is even more blurry from the patients’ perspective.

Importantly, this review reveals that CAs take up a range of roles in clinical and care settings some of which are informally negotiated based on competency levels, years of experience, confidence, or supervision effort required. These insights are similar to observations by Just et al. [63] while examining the role of CAs in end-of-life care and McKenna et al. in their review on how CAs’ roles affect patient safety and care quality. The majority of the CAs’ assigned tasks are direct patient care activities, including vital signs monitoring, assisting with patient hygiene and elimination needs, and support with patient medication—all of which help to meet essential patient care needs. This suggests that with good mentorship, competency-based training, and appropriate supervision, such tasks could be progressively assigned to lower-cadre assistive personnel. Interestingly, we also note that there are higher-level extended roles undertaken by the CAs, namely, basic life support, giving injections, wound care, ECG monitoring, catheterisation, and sample(specimen) collection. However, in support of other studies elsewhere [63, 95], these tasks are mostly informally negotiated, setting-specific, and would require a higher level of skills and training. Notably, the extended roles were common in USA, Canada, UK, and Australia, which could be attributed to deliberate staff upskilling incentives and/or the availability of good on-the-job competency development programmes in these settings. More importantly, clarity of role boundaries and accountability mechanisms that avoid role conflict with the qualified nursing workforce appears to be missing. Overall, the nature of pre-service, and on-the-job training requirements determine the roles and depth to which tasks are assigned and executed by the CAs [24, 40, 45, 60, 64, 81, 96, 97].

How CAs are recruited and deployed in formal care settings has the potential to affect patient care outcomes and shape nurses’ experiences of care delivery. We infer that hospitals choose between two strategies of integration of CAs in the hospital setting: a substitutive model [50, 73] where the CAs are employed to cover the shortage of nursing staff and a supplemental model where the CAs are added as a layer to an existing proportionately ‘optimal’ staff. However, these strategies have only been explicitly reported in the UK and Australia [50, 73, 98]. Still, the choice of either approach is very much inconsistent or unclear across and within most care settings [50, 73]. This could be explored further in future research. Supplementation of nurses with CAs creates an incremental effect on skill-mix and is generally linked with positive patient outcomes and clinical staff experiences, including promoting continuity of quality and effective care through fewer work interruptions, reduced waiting time, and overall, reduction in the risk for missed care. On the other hand, the substitutive strategy is associated with a reduced nurse–patient contact time, leading to concerns about patient safety [14, 69, 71], role deprivation, loss of professional identity, reduced job satisfaction, and unclear accountability lines for actions [53, 63, 95]. Noteworthy, as illustrated in Table 3 and Table 4*,* a few papers had impacts and experiences that conflict with others. For instance, patient safety is not only viewed in a positive sense (i.e., reduced patient injury [48, 54] but equally in a negative sense (i.e., heightened risk to patient safety [42, 50, 68]. Similarly, job satisfaction among nurses has both negative [54] and positive[59, 79] sentiments from different settings. Understandably, as reported elsewhere [99, 100] the effects related to patient safety and satisfaction present potential for medical–legal issues, although this implication was not observed in the current review.

With the ever-increasing strain on healthcare globally, many healthcare systems have been pushed to adopt mechanisms to optimize care delivery amidst limited workforce capacities [43]. Coupled with the slim evidence on specific safe staffing mix ratios, CAs have become a norm in healthcare systems albeit, largely informally in most countries. Although a few studies reported some form of regulatory or clinical governance mechanism, the general picture is a lack of standardised regulatory frameworks for training, employment, degree of task-shifting/task-sharing, the delegation of duties, and supervision and accountability of the CAs. In LMICs, this cadre of staff remains marginalized and or unofficially recognised. Effective management and utilisation of this workforce in healthcare remains disjointed and could lead to either exploitation or underutilisation [43, 73, 85]. Moreover, the lack of clarity on their contribution to patient safety and quality of care begs further investigation. In essence, the adoption and use of CAs in healthcare require a careful approach to sustain professional accountability and avoid over-dilution of skill-mix [42, 94, 97, 101].

The synthesis of the finding of this review is cognisant of some limitations. First, since this is a review, its methodology is unable to make causal inferences on how CAs affect patient care outcomes. However, our findings help to highlight gaps that could be addressed by robust empirical study designs. Second, our eligibility was limited to records in English-language only. However, despite adopting an open search period and across multiple databases and registries, we only retrieved and synthesised literature from 15 countries. This could imply this topic remains under-studied in the countries with no available literature.

## Conclusion

In summary, the nomenclature for CAs is largely inconsistent and variably used. Second, the application of task-shifting strategies in hospitals is fragmented and this is observed both within and across countries. Third, the effect of CAs on patient care outcomes or nurses’ experiences remains unclear as both positive and negative sentiments have been reported in equal measure. Consequently, the synthesis of these findings has several implications. First, we provide evidence that the 2008 version of the International Labour Organization’s International Standard Classification of Occupations [ILO’s ISCO] [92] and related national occupation classifications should be updated for a more clear and more meaningful nomenclature for CAs and other related assistive personnel. Second, collating and mapping empirical evidence on regulatory and clinical governance mechanisms for CAs and their impact on patient care as well as nurses’ experiences present an opportunity to advance conversations on future research on HRH for assistive personnel. For instance, the amount of net savings or costs incurred relative to gains on service delivery and efficiency from assistive personnel in healthcare facilities. Third, these findings bring to the fore, a need for the operationalisation of context-specific policy guidelines and strategies for task-shifting/sharing, including the training of CAs, within a larger framework for norms and standards for HRH management. Such guidance will need to accommodate a diverse and changing landscape of CA roles in hospital settings.

### Supplementary Information

**Additional file 1. **Summary of keywords and synonyms. Keywords and synonyms used in search strategy.

**Additional file 2. **Full search strategy for PUBMED and CINAHL. Sample search strategy for select databases as extended data.

**Additional file 3. **Summary of inclusion and exclusion criteria. Eligibility criteria for screening of sources.

**Additional file 4. **Characteristics of individual sources of evidence and setting. Characteristics of sources of evidence.

**Additional file 5. **Descriptors for ward/care assistants in various countries. Titles used to describe ward/care assistants in various countries.

**Additional file 6. **Cross-cutting titles for Care Assistants. Stem-word title descriptors for care assistants.

**Additional file 7. **Outcome mapping from sources of evidence. Mapping whether sources report on the outcomes of interest.

**Additional file 8. **Regulatory and clinical governance mechanisms. List of regulatory and clinical governance mechanisms as reported from individual countries and sources.

**Additional file 9. **Topics/skills covered during Care Assistants’ training. List of training topics/content.

**Additional file 10. **PRISMA–ScR Checklist. PRISMA checklist extension for scoping reviews.

## Data Availability

No underlying primary data are associated with this study. However, the search strategy as an extended data is available from the Open Science Framework Registries [31]. Any other data are available and shareable under the terms of the Creative Commons International License (CC-BY 4.0).

## Abbreviations

CAs: Care assistants
CINAHL: Cumulative index to nursing and allied health literature
ECG: Electrocardiogram
HRH: Human resources for health
LMICs: Low- and middle-income countries
PRISMA–ScR: Preferred reporting items for systematic reviews and meta-analysis for scoping review
UK: United Kingdom
USA: The United States of America
ILO’s ISCO: International labour organization’s international standard classification of occupations
