# Supplementary material for: Characterising support and care assistants in formal hospital settings: a scoping review
Source: Hum Resour Health. 2023 Nov 27;21:90. doi: 10.1186/s12960-023-00877-7 (PMC10680191; doi:10.1186/s12960-023-00877-7)
Supplement: Supplementary file 1 — Additional file 1. Summary of keywords and synonyms. Keywords and synonyms used in search strategy. [file 12960_2023_877_MOESM1_ESM.docx]

# *Additional file 1: Summary of keywords and synonyms*

| **Keyword** | **Search term/Synonyms** |
| --- | --- |
| **Ward assistant /**  **Care assistant** | - Healthcare assistant OR - Healthcare aide OR - Nursing assistant OR - Care assistant OR - Nurse aide OR - Orderly/Orderlies OR - Nurse auxiliary(ies) OR - Medical assistant OR - Ward assistant/attendant OR - Patient assistant/attendant OR - Ward/Hospital support worker OR - Healthcare support worker OR - Support staff/worker OR - Lay worker OR |
| **Ward clerk** | - Hospital ward clerk OR - Casual OR |
| **Porter** | - Hospital/Ward porter OR |
| **Task-shifting** | - Task shifting OR - Task sharing OR - Task substitution OR - Task delegation OR - Role allocation OR |
|  | **AND** |
| **Patient care** | - Patient safety OR - Patient care OR - Care outcome OR - Quality of care OR - Nursing care OR - Staffing OR - Staffing norm/pattern |
