# Supplementary material for: Characterising support and care assistants in formal hospital settings: a scoping review
Source: Hum Resour Health. 2023 Nov 27;21:90. doi: 10.1186/s12960-023-00877-7 (PMC10680191; doi:10.1186/s12960-023-00877-7)
Supplement: Supplementary file 2 — Additional file 2. Full search strategy for PUBMED and CINAHL. Sample search strategy for select databases as extended data. [file 12960_2023_877_MOESM2_ESM.docx]

# *Additional file 2: Full search strategy for PUBMED and CINAHL*

| **DATABASE** | **SEARCH STRING** |
| --- | --- |
| **PUBMED** | (((Healthcare assistan*[Title/Abstract] OR Healthcare aide[Title/Abstract] OR Nursing Assistan*[Title/Abstract] OR Medical assistan*[Title/Abstract] OR Ward assistan*[Title/Abstract] OR Ward attendan*[Title/Abstract] OR Patient assistan*[Title/Abstract] OR Patient attendan*[Title/Abstract] OR Nurse aide[Title/Abstract] OR Ward support worker[Title/Abstract] OR Hospital support worker[Title/Abstract] OR Healthcare support worker[Title/Abstract] OR Support staff[Title/Abstract] OR Support worker[Title/Abstract] OR Lay worker[Title/Abstract] OR Nurse auxiliar*[Title/Abstract] OR Hospital ward clerk[Title/Abstract] OR Casual[Title/Abstract] OR Hospital porter[Title/Abstract] OR Orderl*[Title/Abstract] OR Task shifting[Title/Abstract] OR Task sharing[Title/Abstract] OR Task substitution[Title/Abstract] OR Task delegation[Title/Abstract] OR Task allocation[Title/Abstract] OR Role allocation[Title/Abstract] OR Paramedical personnel[Title/Abstract]) AND (Patient safety[Title/Abstract] OR Care[Title/Abstract] OR “Quality of care”[Title/Abstract] OR Financ*[Title/Abstract] OR Budget*[Title/Abstract] OR Cost[Title/Abstract] OR Staff*[Title/Abstract] OR [Title/Abstract] OR Extra personnel[Title/Abstract] OR Extra staff[Title/Abstract]))) AND ((humans[Filter]) AND (english[Filter])) |
| **CINAHL** | \| S10 \| S6 AND S9  **Limiters** - English Language; Peer Reviewed; Research Article; Exclude MEDLINE records; Human; Publication Type: Clinical Trial, Corrected Article, Journal Article, Nursing Interventions, Randomized Controlled Trial, Research, Review, Systematic Review; Language: English  **Expanders** - Apply equivalent subjects  **Search modes** - Boolean/Phrase \| \| --- \| --- \| \| S9 \| S7 OR S8 \| \| S8 \| TI staff* OR AB staff* OR TI personnel OR AB personnel \| \| S7 \| TI Patient safety OR AB patient safety OR TI care OR AB care OR TI quality of care OR AB quality of care OR TI financ* OR AB financ* OR TI budget* OR AB budget* OR TI cost OR AB cost \| \| S6 \| S1 OR S2 OR S3 OR S4 OR S5 \| \| S5 \| AB hospital ward clerk OR TI casual OR AB casual \| \| S4 \| TI task sharing OR AB task sharing OR TI task shifting OR TI task substitution OR AB task substitution OR TI ward assistan* OR AB ward assistan* OR TI ward attendan* OR AB ward attendan* OR TI ward support worker OR AB ward support worker OR TI hospital ward clerk \| \| S3 \| TI Role allocation OR AB Role allocation OR TI scrub technician OR TI support staff OR AB support staff OR TI support worker OR AB support worker OR TI task allocation OR AB task allocation OR TI task delegation OR AB task delegation \| \| S2 \| TI nurse auxiliar* OR AB nurse auxiliar* OR TI nursing assistan* OR AB nursing assistan* OR TI orderl* OR AB orderl* OR TI paramedical personnel OR AB paramedical personnel OR TI patient assistan* OR AB patient assistan* OR TI patient attendan* OR AB patient attendan* \| \| S1 \| TI assistant practitioner OR AB assistant practitioner OR TI healthcare assistant OR AB healthcare assistant OR TI health care support worker OR AB healthcare support worker OR TI lay worker OR AB lay worker OR TI medical assistan* OR AB medical assistan* OR TI nurse aide OR AB nurse aide \| |
