# Supplementary material for: Characterising support and care assistants in formal hospital settings: a scoping review
Source: Hum Resour Health. 2023 Nov 27;21:90. doi: 10.1186/s12960-023-00877-7 (PMC10680191; doi:10.1186/s12960-023-00877-7)
Supplement: Supplementary file 3 — Additional file 3. Summary of inclusion and exclusion criteria. Eligibility criteria for screening of sources. [file 12960_2023_877_MOESM3_ESM.docx]

Additional file 3: Summary of inclusion and exclusion criteria

|  | **Inclusion criteria** | **Exclusion criteria** |
| --- | --- | --- |
| **Electronic database/**  **registry** | - PUBMED - Cumulative Index to Nursing and Allied Health Literature (CINAHL) - PsychINFO - EMBASE - Web of Science - Scopus - Google Scholar | - Registry with non-health or medical sciences papers |
| **Participants** | Supportive/Assistive healthcare personnel as defined in this scoping review | - Animal studies - Ward assistants with formal pre-service training, and/or licensing requirements |
| **Context/Setting** | Inpatient and outpatient care settings across the globe | - Community/residential homes |
| **Concepts** | - Patient Care outcomes - Regulatory framework - Clinical governance mechanism - Staffing norms and experiences | - Source not reporting any of the outcomes listed herein |
| **Study designs and type** | - Experimental studies (*randomised and non-randomised*) - Observational studies (*cross-sectional, cohort, case-control, and health economic evaluation*) - Qualitative studies - Mixed methods - Case reports - Grey literature including white papers | - Thesis/Dissertations - Conference abstracts/seminar reports - Commentary/Expert opinions |
| **Time** | No limit on the search period | - |
| **Language** | - Published in English | - Non-English published papers |
