# Supplementary material for: Characterising support and care assistants in formal hospital settings: a scoping review
Source: Hum Resour Health. 2023 Nov 27;21:90. doi: 10.1186/s12960-023-00877-7 (PMC10680191; doi:10.1186/s12960-023-00877-7)
Supplement: Supplementary file 4 — Additional file 4. Characteristics of individual sources of evidence and setting. Characteristics of sources of evidence. [file 12960_2023_877_MOESM4_ESM.docx]

# *Additional file 4: Characteristics of individual sources of evidence and setting*

| No | Author, year,  *(Country)* | Design | Terms used to define describe the HWA | Education level of HWA | Hospital setting (type);  *Hospital department* |
| --- | --- | --- | --- | --- | --- |
| 1 | King et al., 2009  *(UK)* | Case study | Healthcare assistant | # | Urban (Public);  *Children ICU* |
| 2 | Spilsbury et al., 2004  *(UK)* | Case study | Healthcare assistant | Mixed | Urban (Public);  *General Inpatient* |
| 3 | Nyberg et al., 1997  (USA) | Case study | Unlicensed assistive personnel | Secondary  (High school) | Urban (Private);  *Children Operating Room* |
| 4 | TAFE SA, 2022  *(Australia)* | Course guide/Training pack | Nursing assistant, nursing support worker, patient service attendant, patient support assistant, patient care assistant, orderly, theatre support, ward assistant, wards person, ward support | # | Mixed (#);  # |
| 5 | Open Colleges, 2022  *(Australia)* | Course guide/Training pack | Nursing assistant, patient care assistant, patient support assistant, ward assistant, ward support person | # | Mixed (#);  # |
| 6 | City and Guilds, 2022  *(UK)* | Course guide/Training pack | Health and social care worker | # | Mixed (#);  # |
| 7 | Skills for Health, 2020  *(UK)* | Course guide/Training pack | Healthcare assistant, support worker | # | # (Public and Private);  *Both Inpatient and Outpatient* |
| 8 | American Red Cross, 2022  *(USA)* | Course guide/Training pack | Nurse assistant | # | # (#);  # |
| 9 | Roche et al., 2016  *(Australia)* | Cross-sectional | Assistant in Nursing | # | Urban and Rural (Public);  *General Inpatient* |
| 10 | Duffield et al., 2018  *(Australia)* | Cross-sectional | Nursing support worker | # | # (Public);  *General Inpatient* |
| 11 | Peduzzi et al., 2006  *(Brazil)* | Cross-sectional | Nurse assistant, unlicensed nurse | no formal education | Urban and Rural (Public and Private);  *General Inpatient* |
| 12 | Barken et al., 2015  *(Canada)* | Cross-sectional | Home care worker; support worker; ancillary worker; healthcare assistant; home care assistant/aide | # | # (#);  *Both Inpatient and Outpatient* |
| 13 | Griffiths et al., 2016  *(UK)* | Cross-sectional | Healthcare support worker | # | Urban and Rural (Public);  *Medical and Surgical wards* |
| 14 | Omondi et al., 2020  *(Kenya)* | Cross-sectional | Patient attendant, casuals, mothers/caregivers | # | Mixed (Public and Private);  *Paediatric Inpatient* |
| 15 | Tou et al., 2020  *(Taiwan, China)* | Cross-sectional | Nursing aides | Mixed | # (Public and Private);  *Nursing Care Home* |
| 16 | Burns et al., 2007  *(UK)* | Cross-sectional | Healthcare assistant | # | Rural (Public);  *General Outpatient* |
| 17 | Thornley et al., 2000  *(UK)* | Cross-sectional | Healthcare assistant, nursing assistant | Mixed | Mixed (Public and Private);  # |
| 18 | Ward et al., 2014  *(USA)* | Descriptive survey | Certified nursing assistant | # | # (Public and Private);  *Adult Inpatient* |
| 19 | Handschu et al., 1973  *(USA)* | Descriptive survey | Nurse aide | Secondary  (High school) | Urban and Rural (Private);  *Nursing Care Home* |
| 20 | Trinkoff et al., 2017  *(USA)* | Ecological study | Certified nursing assistant | # | # (Private);  *Nursing Care Home* |
| 21 | McMullen et al., 2015  *(USA)* | Explorative survey | Certified nurse aide | # | Urban and Rural (Private);  *Long-Term Care Facility* |
| 22 | Arnon et al., 2018  *(Israel)* | Intervention evaluation | Orderlies, Transport staff | # | Urban (Public);  *Both Inpatient and Outpatient* |
| 23 | Duffield et al., 2019  *(Australia)* | Mixed methods | Nursing assistant | # | # (Public);  *General Inpatient* |
| 24 | Furaker et al., 2008  *(Sweden)* | Mixed methods | Healthcare assistant, mental assistant | Secondary  (High school) | # (Public);  *General Inpatient* |
| 25 | Weir et al., 2015  *(UK)* | Mixed methods | Healthcare assistant | # | # (Public);  *GP Clinics* |
| 26 | Wild et al., 2011  *(UK)* | Mixed methods | Social care support worker | # | Mixed (#);  *Adult Inpatient* |
| 27 | Roche et al., 2017  *(Australia)* | Observational | Assistant in Nursing | # | Urban (Public);  *General Inpatient* |
| 28 | Mallidou et al., 2013  *(Canada)* | Observational | Healthcare aide | # | Urban (Private);  *Residential Care Facility* |
| 29 | McCloskey et al., 2015  *(Canada)* | Observational | Resident attendant/aide | # | Rural (Private);  *Nursing Care Home* |
| 30 | Chang et al., 1995  *(Hong Kong, China)* | Observational | Support worker; ward auxiliary; sister’s assistant; care assistant; nursing technical assistant; nurse extender; patient care technician | Mixed | # (Public);  *General Inpatient* |
| 31 | Hasson et al., 2005  *(Republic of Ireland)* | Observational | Healthcare assistant | # | # (Public);  *Maternity Unit* |
| 32 | Smith et al., 2001  *(USA)* | Observational | Certified nursing assistant, nursing assistant | No formal education | Rural (Public);  # |
| 33 | Cartwright et al., 2021  *(Australia)* | Observational (Prospective) | Medical imaging assistant | # | Urban (Public and Private);  *Medical Imaging Department* |
| 34 | Hirose et al., 2022  *(Japan)* | Observational (Retrospective cohort) | Nurse aide | # | Urban (Public and Private);  *Surgical ward* |
| 35 | Yang et al., 2015  *(Taiwan, China)* | Observational (Retrospective cohort) | Nurse aide | # | Rural (#);  Adult Inpatient |
| 36 | Griffiths et al., 2019  *(UK)* | Observational (Retrospective) | Nursing assistant | # | Urban (Public);  *Medical and Surgical wards* |
| 37 | Faulkner et al., 2016  *(UK)* | Observational cohort | Healthcare assistant | # | # (Public);  *GP Clinics* |
| 38 | Nabudere et al., 2011  *(Uganda)* | Policy brief | Lay health worker | # | # (#);  # |
| 39 | Tzeng et al., 2004  *(Taiwan, China)* | Position paper | Nurse aides, personal attendant, assistant nurse, nursing assistant, ward attendant | Secondary  (High school) | Urban (Public);  *General Inpatient* |
| 40 | The North-West Accident and Emergency Managers' Forum, 1997  *(UK)* | Position paper | Healthcare support worker | # | Urban (Public);  *Adult Outpatient* |
| 41 | Olson et al., 2013  *(Malawi)* | Pre-Post intervention | Vital signs assistant | Secondary  (High school) | Urban (Public);  *Paediatric Inpatient* |
| 42 | Zeytinoglu et al., 2014  *(Canada)* | Qualitative | Personal support worker | # | # (Public);  *Adult Inpatient* |
| 43 | Gransjön Craftman et al., 2016  *(Sweden)* | Qualitative | Unlicensed assistant personnel | # | Urban (Private);  *Nursing Care Home* |
| 44 | Bach et al., 2008  *(UK)* | Qualitative | Health care assistant, nurse auxiliary | Vocational | Urban (Public);  *Adult Inpatient* |
| 45 | Warr et al., 2002  *(UK)* | Qualitative | Healthcare assistant, auxiliary/Nursing auxiliary, support worker | Vocational | Urban (#);  *General Inpatient* |
| 46 | Franzosa et al., 2018  *(USA)* | Qualitative | Home health aide | # | Urban (Private);  *Nursing Care Home* |
| 47 | Spilsbury et al., 2005  *(UK)* | Qualitative | Healthcare assistant | Mixed | Urban (Public);  *Medical and Surgical wards* |
| 48 | Hancock et al., 2006  *(UK)* | Qualitative | Healthcare assistant; nursing auxiliary | # | # (Public);  *Medical Ward* |
| 49 | Francomb et al., 1997  *(UK)* | Qualitative | Support worker; healthcare assistant | # | # (Public);  *Maternity Unit* |
| 50 | Abrahamson et al., 2020  *(USA)* | Qualitative | Nursing assistant | # | Mixed (Private);  *Nursing Care Home* |
| 51 | Jennings et al., 2011  *(Benin)* | Quasi-experimental | Lay nurse aide | no formal education | Rural (Public);  *Maternity Unit* |
| 52 | MacKay et al., 2014  *(Canada)* | Quasi-experimental | Medical office assistant | Vocational | Rural (Private);  *Family Physician Clinic* |
| 53 | Duffield et al., 2014  *(UK)* | Review | Nursing support worker | # | NA;  NA |
| 54 | Vaughan et al., 2014  *(Global)* | Review | Healthcare support worker | # | # (NA);  NA |
| 55 | Walker et al., 2008  *(Global)* | Review | Healthcare support worker | # | NA;  NA |
| 56 | Bosley et al., 2008  *(UK)* | Review | Healthcare assistant | Secondary  (High school) | # (Public);  *Both Inpatient and Outpatient* |
| 57 | McKenna H. et al., 2004  *(Global)* | Review | **US:** nursing assistant, nursing aide, patients care aides, nursing service technicians, unlicensed assistive personnel.  **UK:** generic support worker, healthcare assistant, clinical support worker, ward assistant, care worker, home care assistant, bed maker | Mixed | # (NA);  NA |
| 58 | Hewko et al., 2015  *(Global)* | Scoping review | Healthcare aide | Mixed | Mixed (Public and Private);  *Both Inpatient and Outpatient* |
| 59 | Just et al., 2021  *(Global)* | Scoping review | Healthcare assistant, personal support worker, nursing assistant | # | # (Public and Private);  *End of Life Care Facility* |
| 61 | Hyer et al., 2011  *(USA)* | Survey | Certified nursing assistant | # | # (Private);  *Nursing Care Home* |
| 62 | Castle et al., 2011  *(USA)* | Survey | Nurse aide | Secondary  (High school) | Urban and Rural (Private);  *Nursing Care Home* |
| 63 | Gould et al., 1996  *(USA)* | Survey | Nursing assistant | # | Urban (Private);  *Surgical ward* |
| 64 | Blay et al., 2020  *(Global)* | Systematic review | Nursing assistant, USA: Unlicensed assistive personnel, nursing aide; UK: healthcare assistant; New Zealand: nurse assistant; Australia: Assistant in nursing | # | Mixed (Public and Private);  *Both Inpatient and Outpatient* |
| 65 | WHO OptimizeMNH, 2014  *(Global)* | White paper | Auxiliary nurse midwife, lay health worker, community health worker, traditional birth attendant | Secondary  (High school) | Mixed (#);  # |
| 66 | WHO OptimizeMNH, 2012  *(Global)* | White paper | Auxiliary nurse, nurse assistant, auxiliary nurse midwife, auxiliary midwife, lay health worker | Secondary  (High school) | Mixed (#);  # |
| 67 | WHO, 2008  *(Global)* | White paper | Auxiliary nurse/assistant | # | Mixed (#);  # |
| 68 | KWTRP, 2018  *(Kenya)* | White paper | Newborn healthcare assistant | Mixed | Urban (Public);  *Paediatric Inpatient* |
| 69 | Ministry of Health, 2014  *(Kenya)* | White paper | Nurse assistant; patient attendant; casual; support staff | # | Mixed (Public);  *Both Inpatient and Outpatient* |
| 71 | National Health Service, 2024  *(UK)* | White paper | Healthcare assistant | # | # (Public);  *Both Inpatient and Outpatient* |
| 72 | Cavendish et al., 2013  *(UK)* | White Paper | Healthcare assistant, support worker | # | Mixed (#);  # |
| 73 | Arblaster et al., 2004  *(UK)* | White paper | Healthcare support worker | # | Urban and Rural (Public and Private);  # |
| 74 | National Health Service, 2023  *(UK)* | White paper | Healthcare support worker | # | # (Public);  *Both Inpatient and Outpatient* |
| 75 | National Council of State Boards of Nursing, 2016  *(USA)* | White Paper | Unlicensed assistive personnel, certified nursing assistant, certified nursing aide | # | Mixed (#);  # |
| # - Not reported in the paper \| NA - Not Applicable | | | | | |
