# Supplementary material for: Characterising support and care assistants in formal hospital settings: a scoping review
Source: Hum Resour Health. 2023 Nov 27;21:90. doi: 10.1186/s12960-023-00877-7 (PMC10680191; doi:10.1186/s12960-023-00877-7)
Supplement: Supplementary file 5 — Additional file 5. Descriptors for ward/care assistants in various countries. Titles used to describe ward/care assistants in various countries. [file 12960_2023_877_MOESM5_ESM.docx]

# *Additional file 5: Descriptors for ward/care assistants in various countries*

|  | Region (Income-group) | Country | Descriptors for Ward Assistants |
| --- | --- | --- | --- |
| 1 | Western Pacific  (HIC) | Australia(1, 2, 3, 4, 5, 6, 7, 8) | Assistant In Nursing [AIN]; Medical Imaging Assistant; Nursing Assistant; Nursing Support Worker; Patient Service Attendant; Patient Support Assistant; Patient Care Assistant; Orderly, Theatre Support; Ward Assistant; Ward Support Person |
| 2 |  | Hong Kong; China(9) | Support Worker; Ward Auxiliary; Sister's Assistant; Care Assistant; Nursing Technical Assistant; Nurse Extender; Patient Care Technician |
| 3 |  | Taiwan, China(10, 11, 12) | Nurse Aide; Personal Attendant; Assistant Nurse; Nursing Assistant; Ward Attendant; Nursing Aide |
| 4 |  | Japan(13) | Nurse Aide |
| 5 |  | New Zealand(1) | Nurse Assistant |
| 6 | South America (UMIC) | Brazil(14) | Nurse Aide; Nurse Assistant; Unlicensed Nurse |
| 7 | Northern America  (HIC) | Canada(15, 16, 17, 18, 19) | Support Worker; Resident Attendant; Resident Aide; Healthcare Aide; Home Care Worker; Ancillary Worker; Healthcare Assistant; Home Care Assistant; Home care Aide; Medical Office Assistant; Nurse Aide |
| 8 |  | USA(20, 21, 22, 23, 24, 25, 26, 27, 28, 29, 30, 31, 32, 33) | Unlicensed Assistive Personnel [UAP]; Certified Nursing Assistant; Certified Nursing Aide; Certified Nurse Aide; Home Health Aide; Patient Care Aide; Nursing Service Technician |
| 9 | European (HIC) | Israel(34) | Orderlies; Transport Staff |
| 10 |  | Republic of Ireland(35) | Healthcare Assistant; Orderlies |
| 11 |  | Sweden(36, 37) | Unlicensed Assistant Personnel; Healthcare Assistant, Mental Assistant |
| 12 |  | United Kingdom(38, 39, 40, 41, 42, 43, 44, 45, 46, 47, 48, 49, 50, 51, 52, 53, 54, 55, 56, 57, 58, 59, 60) | Social Care Support Worker; Support Worker; Healthcare Assistant [HCA]; Health and Social Care Worker; Nurse Auxiliary; Nursing Auxiliary; Support Worker; Nursing Assistant; Healthcare Support Worker; Nursing Support Worker; Clinical Support Worker; Ward Assistant; Care Worker; Home Care Assistant; Bed Maker; Auxiliary Nurse-Midwife; Lay Health Worker; Community Health Worker; Traditional Birth Attendant; Auxiliary Assistant; Healthcare Aide |
| 13 | African Region (LMIC) | Benin(61) | Lay Nurse Aide |
| 14 |  | Kenya(62, 63, 64) | Patient Attendant; Casual worker; Caregivers; Newborn Healthcare Assistant; Nurse Assistant; Support Staff |
| 15 | African Region (LIC) | Malawi(65) | Vital Signs Assistant |
| 16 |  | Uganda(66) | Lay Health Worker |
|  | **NB:** HIC – High-Income Country **\|** UMIC – Upper Middle-Income Country **\|** LMIC – Lower Middle-Income Country **\|** LIC – Low-Income Country | | |

# **References**

1. Blay N, Roche MA. A systematic review of activities undertaken by the unregulated Nursing Assistant. J Adv Nurs. 2020;76(7):1538-51.

2. Cartwright AK, Pain T, Heslop DJ. Substitution, delegation or addition? Implications of workforce skill mix on efficiency and interruptions in computed tomography. Aust Health Rev. 2021;45(3):382-8.

3. Duffield C, Roche M, Twigg D, Williams A, Rowbotham S, Clarke S. Adding unregulated nursing support workers to ward staffing: Exploration of a natural experiment. J Clin Nurs. 2018;27(19-20):3768-79.

4. Duffield C, Twigg D, Roche M, Williams A, Wise S. Uncovering the Disconnect Between Nursing Workforce Policy Intentions, Implementation, and Outcomes: Lessons Learned From the Addition of a Nursing Assistant Role. Policy Polit Nurs Pract. 2019;20(4):228-38.

5. Roche MA, Duffield C, Friedman S, Dimitrelis S, Rowbotham S. Regulated and unregulated nurses in the acute hospital setting: Tasks performed, delayed or not completed. Journal of Clinical Nursing (John Wiley & Sons, Inc). 2016;25(1-2):153-62.

6. Roche MA, Friedman S, Duffield C, Twigg DE, Cook R. A comparison of nursing tasks undertaken by regulated nurses and nursing support workers: a work sampling study. J Adv Nurs. 2017;73(6):1421-32.

7. Open Colleges. Should you work as a ward assistant? opencolleges.edu.au: Open Colleges; 2019 [Available from: <https://www.opencolleges.edu.au/careers/blog/should-you-work-ward-assistant#:~:text=Ward%20assistants%20perform%20the%20day,that%20keep%20a%20ward%20running>.

8. TAFE South Australia. Certificate III in Allied Health Assistance online: TAFE SA; 2022 [cited 2023 April 2023]. Available from: <https://www.tafesa.edu.au/xml/course/aw/aw_TP00870.aspx?S=AWD&Y=2023>.

9. Chang AM. Perceived functions and usefulness of health service support workers. J Adv Nurs. 1995;21(1):64-74.

10. Tou YH, Liu MF, Chen SR, Lee PH, Kuo LM, Lin PC. Investigating missed care by nursing aides in Taiwanese long‐term care facilities. Journal of Nursing Management (John Wiley & Sons, Inc). 2020;28(8):1918-28.

11. Tzeng HM. Roles of nurse aides and family members in acute patient care in Taiwan. Journal of Nursing Care Quality. 2004;19(2):169-75.

12. Yang PH, Hung CH, Chen YC. The impact of three nursing staffing models on nursing outcomes. J Adv Nurs. 2015;71(8):1847-56.

13. Hirose N, Morita K, Matsui H, Fushimi K, Yasunaga H. Association between nurse aide staffing and patient mortality after major cancer surgeries in acute care settings: A retrospective cohort study. Nurs Health Sci. 2022;24(1):283-92.

14. Peduzzi M, Anselmi ML, França Jr I, dos Santos CB. Quality of procedures delivered by nursing assistants. Rev Saude Publica. 2006;40(5):843-50.

15. Barken R, Denton M, Plenderleith J, Zeytinoglu IU, Brookman C. Home Care Workers' Skills in the Context of Task Shifting: Complexities in Care Work. Can Rev Sociol. 2015;52(3):289-309.

16. MacKay FD, Anderson JE, Klein MC, Berkowitz J, MacKay JT, Gailius J. The modified medical office assistant role in rural diabetes care. Canadian Journal of Rural Medicine (Joule Inc). 2014;19(2):49-56.

17. Mallidou AA, Cummings GG, Schalm C, Estabrooks CA. Health care aides use of time in a residential long-term care unit: A time and motion study. International Journal of Nursing Studies. 2013;50(9):1229-39.

18. McCloskey R, Donovan C, Stewart C, Donovan A. How registered nurses, licensed practical nurses and resident aides spend time in nursing homes: An observational study. International Journal of Nursing Studies. 2015;52(9):1475-83.

19. Zeytinoglu IU, Denton M, Brookman C, Plenderleith J. Task shifting policy in Ontario, Canada: Does it help personal support workers’ intention to stay? Health Policy. 2014;117(2):179-86.

20. Abrahamson K, Fox R, Roundtree A, Farris K. Nursing assistants' perceptions of their role in the resident experience. Nursing & Health Sciences. 2020;22(1):72-81.

21. American Red Cross. Certified Nursing Assistant2022 January 2023 [cited 2023 January 2023]. Available from: <https://www.redcross.org/take-a-class/cna>.

22. Castle NG, Anderson RA. Caregiver staffing in nursing homes and their influence on quality of care: using dynamic panel estimation methods. Med Care. 2011;49(6):545-52.

23. Franzosa E, Tsui EK, Baron S. Home Health Aides' Perceptions of Quality Care: Goals, Challenges, and Implications for a Rapidly Changing Industry. New Solut. 2018;27(4):629-47.

24. Gould R, Thompson R, Rakel B, Jensen J, Hasselman E, Young L. Redesigning the RN and NA roles. Nursing Management. 1996;27(2):37-43.

25. Handschu SS. Profile of the nurse’s aide expanding her role as psycho-social companion to the nursing home resident. Gerontologist. 1973;13(3):315-7.

26. Hyer K, Thomas KS, Branch LG, Harman JS, Johnson CE, Weech-Maldonado R. The influence of nurse staffing levels on quality of care in nursing homes. Gerontologist. 2011;51(5):610-6.

27. McKenna HP, Hasson F, Keeney S. Patient safety and quality of care: the role of the health care assistant. J Nurs Manage. 2004;12(6):452-9.

28. McMullen TL, Resnick B, Chin-Hansen J, Geiger-Brown JM, Miller N, Rubenstein R. Certified Nurse Aide Scope of Practice: State-by-State Differences in Allowable Delegated Activities. Journal of the American Medical Directors Association. 2015;16(1):20-4.

29. National Council of State Boards of Nursing. National Guidelines for Nursing Delegation. Journal of Nursing Regulation. 2016;7(1):5-14.

30. Nyberg DB, Campbell JL. An orientation program for unlicensed assistive personnel. AORN journal. 1997;66(3):445-9, 52-54.

31. Smith DA. Aide for a day. Journal of the American Medical Directors Association. 2001;2(4):166-9.

32. Trinkoff AM, Storr CL, Lerner NB, Yang BK, Han K. CNA training requirements and resident care outcomes in nursing homes. Gerontologist. 2017;57(3):501-8.

33. Ward S, Stewart D, Ford D, Mullen AM, Makic MBF. Educating certified nursing assistants educational offerings on the run and more. Journal for Nurses in Professional Development. 2014;30(6):296-302.

34. Arnon Z, Ben-Arye E, Attias S, Levy O, Schiff E. Integrative medicine as a change agent of hospital staff: From hospital orderlies to partners in health promotion. European Journal of Integrative Medicine. 2018;18:42-6.

35. Hasson F, McKenna H, Keeney S, Gillen P. What do midwifery healthcare assistants do? Investigating the role of the trained healthcare assistant. RCM Midwives. 2005;8(2):74-7.

36. Furaker C. Health care assistants' and mental attendants' daily work tasks in acute hospital care. Journal of Research in Nursing. 2008;13(6):542-53.

37. Gransjön Craftman Å, Grape C, Ringnell K, Westerbotn M. Registered nurses' experience of delegating the administration of medicine to unlicensed personnel in residential care homes. Journal of Clinical Nursing (John Wiley & Sons, Inc). 2016;25(21-22):3189-98.

38. Arblaster G, Streather C, Hugill L, McKenzie M, Missenden J. A training programme for healthcare support workers. Nurs Stand. 2004;18(43):33-7.

39. Bach S, Kessler I, Heron P. Role redesign in a modernised NHS: the case of health care assistants. Human Resource Management Journal. 2008;18(2):171-87.

40. Bosley S, Dale J. Healthcare assistants in general practice: practical and conceptual issues of skill-mix change. British Journal of General Practice. 2008;58(547):118.

41. Burns S, Blair V. Health care assistants in general practice. Primary Health Care. 2007;17(6):35-9.

42. Cavendish C. The Cavendish Review: An Independent Review into Healthcare Assistants and Support Workers in the NHS and social care settings. online: Department of Health and Social Care; 2013 10 July 2013.

43. City and Guilds. City & Guilds RQF (NVQ) Level 2 Diploma in Health & Social Care online: City & Guilds; 2022 [cited 2023 April 2023]. Course guide]. Available from: <https://www.cityandguilds.com/qualifications-and-apprenticeships/health-and-social-care#fil=uk>.

44. Duffield CM, Twigg DE, Pugh JD, Evans G, Dimitrelis S, Roche MA. The Use of Unregulated Staff: Time for Regulation? Policy Polit Nurs Pract. 2014;15(1-2):42-8.

45. Faulkner K, Sutton S, Jamison J, Sloan M, Boase S, Naughton F. Are Nurses and Auxiliary Healthcare Workers Equally Effective in Delivering Smoking Cessation Support in Primary Care? Nicotine Tob Res. 2016;18(5):1054-60.

46. Griffiths P, Ball J, Murrells T, Jones S, Rafferty AM. Registered nurse, healthcare support worker, medical staffing levels and mortality in English hospital trusts: a cross-sectional study. BMJ Open. 2016;6(2):e008751.

47. Griffiths P, Maruotti A, Recio Saucedo A, Redfern OC, Ball JE, Briggs J, et al. Nurse staffing, nursing assistants and hospital mortality: retrospective longitudinal cohort study. BMJ Qual Saf. 2019;28(8):609-17.

48. Hancock H, Campbell S. Developing the role of the healthcare assistant. Nursing standard (Royal College of Nursing (Great Britain) : 1987). 2006;20(49):35-41.

49. King P, Crawford D. Healthcare assistants in the children's intensive care unit. Paediatric nursing. 2009;21(1):48-51.

50. National Health Service. Healthcare Assistant <https://www.healthcareers.nhs.uk/explore-roles/healthcare-support-worker/roles-healthcare-support-worker/healthcare-assistant>: NHS; n.d [cited 2023 February 2023].

51. National Health Service. Healthcare support worker <https://www.healthcareers.nhs.uk/explore-roles/healthcare-support-worker/roles-healthcare-support-worker/healthcare-support-worker>: NHS; n.d [cited 2023 February 2023].

52. Skills for Health, Health Education England, Skills for Care. The Care Certificate Overview. The Care Certificate. <https://www.skillsforhealth.org.uk/info-hub/the-care-certificate/>: Skills for Health; 2020.

53. Spilsbury K, Meyer J. Use, misuse and non-use of health care assistants: understanding the work of health care assistants in a hospital setting. J Nurs Manage. 2004;12(6):411-8.

54. Spilsbury K, Meyer J. Making claims on nursing work: exploring the work of healthcare assistants and the implications for registered nurses' roles. Journal of Research in Nursing. 2005;10(1):65-83.

55. The North-West Accident and Emergency Managers' Forum. Role of the health care support worker in the A & E department. The North-West Accident and Emergency Managers' Forum. Accid Emerg Nurs. 1997;5(3):131-3.

56. Thornley C. A question of competence? Re-evaluatirig the roles of the nursing auxiliary and health care assistant in the NHS. Journal of Clinical Nursing. 2000;9(3):451-8.

57. Vaughan S, Melling K, O'Reilly L, Cooper D. Understanding the debate around regulation of support workers. British Journal of Nursing. 2014;23(5):260-3.

58. Warr J. Experiences and perceptions of newly prepared Health Care Assistants (Level 3 NVQ). Nurse Education Today. 2002;22(3):241-50.

59. Weir J. Effect of a training programme on the work of GP-based HCAs. Practice Nursing. 2015;26(7):351-5.

60. Wild D, Szczepura A, Nelson S. How social care staff working in residential homes perceive their professional status. Nursing Older People. 2011;23(7):29-35.

61. Jennings L, Yebadokpo AS, Affo J, Agbogbe M, Tankoano A. Task shifting in maternal and newborn care: a non-inferiority study examining delegation of antenatal counseling to lay nurse aides supported by job aids in Benin. Implementation science : IS. 2011;6:2.

62. Health Services Unit-KEMRI Wellcome Trust. Draft Report on Scope of work for Neonatal Healthcare Assistants. KEMRI Wellcome Trust; 2018 2018.

63. Ministry of Health Kenya. Kenya Health Workforce Report: The Status of Healthcare Professionals in Kenya, 2015. In: MOH, editor. Nairobi: The Task Force for Global Health; 2015.

64. Omondi GB, Murphy GAV, Jackson D, Brownie S, English M, Gathara D. Informal task-sharing practices in inpatient newborn settings in a low-income setting—A task analysis approach. Nurs Open. 2020;7(3):869-78.

65. Olson D, Preidis GA, Milazi R, Spinler JK, Lufesi N, Mwansambo C, et al. Task shifting an inpatient triage, assessment and treatment programme improves the quality of care for hospitalised Malawian children. Tropical Medicine and International Health. 2013;18(7)(7):879-86.

66. Nabudere H, Asiimwe D, Mijumbi R. Task shifting in maternal and child health care: An evidence brief for Uganda. International Journal of Technology Assessment in Health Care. 2011;27(2):173-9.
