# Supplementary material for: Characterising support and care assistants in formal hospital settings: a scoping review
Source: Hum Resour Health. 2023 Nov 27;21:90. doi: 10.1186/s12960-023-00877-7 (PMC10680191; doi:10.1186/s12960-023-00877-7)
Supplement: Supplementary file 6 — Additional file 6. Cross-cutting titles for Care Assistants. Stem-word title descriptors for care assistants. [file 12960_2023_877_MOESM6_ESM.docx]

# *Additional file 6: Cross-cutting titles for Care Assistants*

| Order | Common Descriptor  (stem word) | Variations |
| --- | --- | --- |
| 1 | **Assistant** | Care Assistant: |
|  |  | Care Assistant(1) |
|  |  | Healthcare Assistant [HCA](2, 3, 4, 5, 6, 7, 8, 9, 10, 11, 12, 13, 14, 15, 16, 17, 18, 19, 20, 21, 22, 23) |
|  |  | Homecare Assistant(5, 18) |
|  |  | Aide: |
|  |  | Nurse Aide(18, 20, 24, 25, 26, 27, 28, 29, 30, 31, 32) |
|  |  | Home Care Aide(5, 33) |
|  |  | Healthcare Aide(34, 35) |
|  |  | Patient Care Aide(18) |
|  |  | Resident Aide(36) |
|  |  | Nursing Assistant(6, 18, 19, 20, 29, 32, 37, 38, 39, 40, 41, 42, 43, 44, 45, 46, 47, 48, 49) |
|  |  | Unlicensed Assistive Personnel [UAP](18, 20, 32, 50, 51) |
|  |  | Ward Assistant(18, 29, 37, 38) |
|  |  | Assistant in Nursing (AIN)(20, 29, 52, 53) |
|  |  | Auxiliary Assistant(54) |
|  |  | Imaging Assistant(55) |
|  |  | Medical Office Assistant(55, 56) |
|  |  | Mental Assistant(8) |
|  |  | Nursing Technical Assistant(1) |
|  |  | Patient Care Assistant(37, 38) |
|  |  | Patient Support Assistant(38) |
|  |  | Sister’s Assistant(1) |
|  |  | Vital Signs Assistant(57) |
| 2 | **Support Staff/Worker** | Support Worker(1, 4, 5, 13, 16, 18, 19, 23, 49, 58, 59, 60, 61) |
|  |  | Ancillary Worker(5) |
|  |  | Bed Maker(18) |
|  |  | Care Worker(18) |
|  |  | Casual Worker(62) |
|  |  | Health and Social Care Worker(58, 63, 64, 65, 66, 67, 68) |
|  |  | Nursing Support Worker(37, 52) |
|  |  | Patient Support Assistant(37) |
|  |  | Theatre Support Staff(37) |
|  |  | Ward Support Person(37, 38) |
|  |  | Lay Health Worker(69, 70) |
|  |  | Community Health Worker(70) |
|  |  | Home Care Worker(5) |
| 3 | **Auxiliary Staff** | Nurse Auxiliary(12, 13, 15, 70) |
|  |  | Auxiliary Assistant(54, 70) |
|  |  | Auxiliary Attendant[13](#_ENREF_13), [89](#_ENREF_89) |
|  |  | Unlicensed Nurse(40) |
|  |  | Ward Auxiliary(1) |
|  |  | Auxiliary midwife(70) |
| 4 | **Attendant** | Patient Service Attendant(29, 37, 49, 62) |
|  |  | Resident Attendant(29, 36) |
|  |  | Traditional Birth Attendant(70) |
| 5 | **Orderly** | Orderly(37, 71) |
| 6 | **Technician** | Nursing Service Technician(18) |
|  |  | Patient Care Technician(1) |
| 7 | **Extender** | Nurse Extender(1) |
| 8 | **Porter** | Transport Staff(71) |
|  |  | Porter(71) |

# **References**

1. Chang AM. Perceived functions and usefulness of health service support workers. J Adv Nurs. 1995;21(1):64-74.

2. King P, Crawford D. Healthcare assistants in the children's intensive care unit. Paediatric nursing. 2009;21(1):48-51.

3. Spilsbury K, Meyer J. Use, misuse and non-use of health care assistants: understanding the work of health care assistants in a hospital setting. J Nurs Manage. 2004;12(6):411-8.

4. Skills for Health, Health Education England, Skills for Care. The Care Certificate Overview. The Care Certificate. <https://www.skillsforhealth.org.uk/info-hub/the-care-certificate/>: Skills for Health; 2020.

5. Barken R, Denton M, Plenderleith J, Zeytinoglu IU, Brookman C. Home Care Workers' Skills in the Context of Task Shifting: Complexities in Care Work. Can Rev Sociol. 2015;52(3):289-309.

6. Thornley C. A question of competence? Re-evaluatirig the roles of the nursing auxiliary and health care assistant in the NHS. Journal of Clinical Nursing. 2000;9(3):451-8.

7. Burns S, Blair V. Health care assistants in general practice. Primary Health Care. 2007;17(6):35-9.

8. Furaker C. Health care assistants' and mental attendants' daily work tasks in acute hospital care. Journal of Research in Nursing. 2008;13(6):542-53.

9. Weir J. Effect of a training programme on the work of GP-based HCAs. Practice Nursing. 2015;26(7):351-5.

10. Hasson F, McKenna H, Keeney S, Gillen P. What do midwifery healthcare assistants do? Investigating the role of the trained healthcare assistant. RCM Midwives. 2005;8(2):74-7.

11. Faulkner K, Sutton S, Jamison J, Sloan M, Boase S, Naughton F. Are Nurses and Auxiliary Healthcare Workers Equally Effective in Delivering Smoking Cessation Support in Primary Care? Nicotine Tob Res. 2016;18(5):1054-60.

12. Bach S, Kessler I, Heron P. Role redesign in a modernised NHS: the case of health care assistants. Human Resource Management Journal. 2008;18(2):171-87.

13. Warr J. Experiences and perceptions of newly prepared Health Care Assistants (Level 3 NVQ). Nurse Education Today. 2002;22(3):241-50.

14. Spilsbury K, Meyer J. Making claims on nursing work: exploring the work of healthcare assistants and the implications for registered nurses' roles. Journal of Research in Nursing. 2005;10(1):65-83.

15. Hancock H, Campbell S. Developing the role of the healthcare assistant. Nursing standard (Royal College of Nursing (Great Britain) : 1987). 2006;20(49):35-41.

16. Francomb H. Do we need support workers in the maternity services? British Journal of Midwifery. 1997;5(11):672-6.

17. Bosley S, Dale J. Healthcare assistants in general practice: practical and conceptual issues of skill-mix change. British Journal of General Practice. 2008;58(547):118.

18. McKenna HP, Hasson F, Keeney S. Patient safety and quality of care: the role of the health care assistant. J Nurs Manage. 2004;12(6):452-9.

19. Just DT, O'Rourke HM, Berta WB, Variath C, Cranley LA. Expanding the Concept of End-of-life Care in Long-term Care: A Scoping Review Exploring the Role of Healthcare Assistants. International journal of older people nursing. 2021;16(2)(2):e12353.

20. Blay N, Roche MA. A systematic review of activities undertaken by the unregulated Nursing Assistant. J Adv Nurs. 2020;76(7):1538-51.

21. Health Services Unit-KEMRI Wellcome Trust. Draft Report on Scope of work for Neonatal Healthcare Assistants. KEMRI Wellcome Trust; 2018 2018.

22. National Health Service. Healthcare Assistant <https://www.healthcareers.nhs.uk/explore-roles/healthcare-support-worker/roles-healthcare-support-worker/healthcare-assistant>: NHS; n.d [cited 2023 February 2023].

23. Cavendish C. The Cavendish Review: An Independent Review into Healthcare Assistants and Support Workers in the NHS and social care settings. online: Department of Health and Social Care; 2013 10 July 2013.

24. Tou YH, Liu MF, Chen SR, Lee PH, Kuo LM, Lin PC. Investigating missed care by nursing aides in Taiwanese long‐term care facilities. Journal of Nursing Management (John Wiley & Sons, Inc). 2020;28(8):1918-28.

25. Handschu SS. Profile of the nurse’s aide expanding her role as psycho-social companion to the nursing home resident. Gerontologist. 1973;13(3):315-7.

26. McMullen TL, Resnick B, Chin-Hansen J, Geiger-Brown JM, Miller N, Rubenstein R. Certified Nurse Aide Scope of Practice: State-by-State Differences in Allowable Delegated Activities. Journal of the American Medical Directors Association. 2015;16(1):20-4.

27. Hirose N, Morita K, Matsui H, Fushimi K, Yasunaga H. Association between nurse aide staffing and patient mortality after major cancer surgeries in acute care settings: A retrospective cohort study. Nurs Health Sci. 2022;24(1):283-92.

28. Yang PH, Hung CH, Chen YC. The impact of three nursing staffing models on nursing outcomes. J Adv Nurs. 2015;71(8):1847-56.

29. Tzeng HM. Roles of nurse aides and family members in acute patient care in Taiwan. Journal of Nursing Care Quality. 2004;19(2):169-75.

30. Jennings L, Yebadokpo AS, Affo J, Agbogbe M, Tankoano A. Task shifting in maternal and newborn care: a non-inferiority study examining delegation of antenatal counseling to lay nurse aides supported by job aids in Benin. Implementation science : IS. 2011;6:2.

31. Castle NG, Anderson RA. Caregiver staffing in nursing homes and their influence on quality of care: using dynamic panel estimation methods. Med Care. 2011;49(6):545-52.

32. National Council of State Boards of Nursing. National Guidelines for Nursing Delegation. Journal of Nursing Regulation. 2016;7(1):5-14.

33. Franzosa E, Tsui EK, Baron S. Home Health Aides' Perceptions of Quality Care: Goals, Challenges, and Implications for a Rapidly Changing Industry. New Solut. 2018;27(4):629-47.

34. Mallidou AA, Cummings GG, Schalm C, Estabrooks CA. Health care aides use of time in a residential long-term care unit: A time and motion study. International Journal of Nursing Studies. 2013;50(9):1229-39.

35. Hewko SJ, Cooper SL, Huynh H, Spiwek TL, Carleton HL, Reid S, et al. Invisible no more: a scoping review of the health care aide workforce literature. BMC Nursing. 2015;14(1):38.

36. McCloskey R, Donovan C, Stewart C, Donovan A. How registered nurses, licensed practical nurses and resident aides spend time in nursing homes: An observational study. International Journal of Nursing Studies. 2015;52(9):1475-83.

37. TAFE South Australia. Certificate III in Allied Health Assistance online: TAFE SA; 2022 [cited 2023 April 2023]. Available from: <https://www.tafesa.edu.au/xml/course/aw/aw_TP00870.aspx?S=AWD&Y=2023>.

38. Open Colleges. Should you work as a ward assistant? opencolleges.edu.au: Open Colleges; 2019 [Available from: <https://www.opencolleges.edu.au/careers/blog/should-you-work-ward-assistant#:~:text=Ward%20assistants%20perform%20the%20day,that%20keep%20a%20ward%20running>.

39. American Red Cross. Certified Nursing Assistant2022 January 2023 [cited 2023 January 2023]. Available from: <https://www.redcross.org/take-a-class/cna>.

40. Peduzzi M, Anselmi ML, França Jr I, dos Santos CB. Quality of procedures delivered by nursing assistants. Rev Saude Publica. 2006;40(5):843-50.

41. Ward S, Stewart D, Ford D, Mullen AM, Makic MBF. Educating certified nursing assistants educational offerings on the run and more. Journal for Nurses in Professional Development. 2014;30(6):296-302.

42. Trinkoff AM, Storr CL, Lerner NB, Yang BK, Han K. CNA training requirements and resident care outcomes in nursing homes. Gerontologist. 2017;57(3):501-8.

43. Duffield C, Twigg D, Roche M, Williams A, Wise S. Uncovering the Disconnect Between Nursing Workforce Policy Intentions, Implementation, and Outcomes: Lessons Learned From the Addition of a Nursing Assistant Role. Policy Polit Nurs Pract. 2019;20(4):228-38.

44. Smith DA. Aide for a day. Journal of the American Medical Directors Association. 2001;2(4):166-9.

45. Griffiths P, Maruotti A, Recio Saucedo A, Redfern OC, Ball JE, Briggs J, et al. Nurse staffing, nursing assistants and hospital mortality: retrospective longitudinal cohort study. BMJ Qual Saf. 2019;28(8):609-17.

46. Abrahamson K, Fox R, Roundtree A, Farris K. Nursing assistants' perceptions of their role in the resident experience. Nursing & Health Sciences. 2020;22(1):72-81.

47. Hyer K, Thomas KS, Branch LG, Harman JS, Johnson CE, Weech-Maldonado R. The influence of nurse staffing levels on quality of care in nursing homes. Gerontologist. 2011;51(5):610-6.

48. Gould R, Thompson R, Rakel B, Jensen J, Hasselman E, Young L. Redesigning the RN and NA roles. Nursing Management. 1996;27(2):37-43.

49. Ministry of Health Kenya. Kenya Health Workforce Report: The Status of Healthcare Professionals in Kenya, 2015. In: MOH, editor. Nairobi: The Task Force for Global Health; 2015.

50. Nyberg DB, Campbell JL. An orientation program for unlicensed assistive personnel. AORN journal. 1997;66(3):445-9, 52-54.

51. Gransjön Craftman Å, Grape C, Ringnell K, Westerbotn M. Registered nurses' experience of delegating the administration of medicine to unlicensed personnel in residential care homes. Journal of Clinical Nursing (John Wiley & Sons, Inc). 2016;25(21-22):3189-98.

52. Roche MA, Duffield C, Friedman S, Dimitrelis S, Rowbotham S. Regulated and unregulated nurses in the acute hospital setting: Tasks performed, delayed or not completed. Journal of Clinical Nursing (John Wiley & Sons, Inc). 2016;25(1-2):153-62.

53. Roche MA, Friedman S, Duffield C, Twigg DE, Cook R. A comparison of nursing tasks undertaken by regulated nurses and nursing support workers: a work sampling study. J Adv Nurs. 2017;73(6):1421-32.

54. World Health Organisation. Task Shifting: Global Recommendations and Guidelines. In: Services HSa, editor. Geneva, Switzerland: WHO Document Production Services; 2008. p. 80.

55. Cartwright AK, Pain T, Heslop DJ. Substitution, delegation or addition? Implications of workforce skill mix on efficiency and interruptions in computed tomography. Aust Health Rev. 2021;45(3):382-8.

56. MacKay FD, Anderson JE, Klein MC, Berkowitz J, MacKay JT, Gailius J. The modified medical office assistant role in rural diabetes care. Canadian Journal of Rural Medicine (Joule Inc). 2014;19(2):49-56.

57. Olson D, Preidis GA, Milazi R, Spinler JK, Lufesi N, Mwansambo C, et al. Task shifting an inpatient triage, assessment and treatment programme improves the quality of care for hospitalised Malawian children. Tropical Medicine and International Health. 2013;18(7)(7):879-86.

58. Wild D, Szczepura A, Nelson S. How social care staff working in residential homes perceive their professional status. Nursing Older People. 2011;23(7):29-35.

59. Zeytinoglu IU, Denton M, Brookman C, Plenderleith J. Task shifting policy in Ontario, Canada: Does it help personal support workers’ intention to stay? Health Policy. 2014;117(2):179-86.

60. Duffield CM, Twigg DE, Pugh JD, Evans G, Dimitrelis S, Roche MA. The Use of Unregulated Staff: Time for Regulation? Policy Polit Nurs Pract. 2014;15(1-2):42-8.

61. Arblaster G, Streather C, Hugill L, McKenzie M, Missenden J. A training programme for healthcare support workers. Nurs Stand. 2004;18(43):33-7.

62. Omondi GB, Murphy GAV, Jackson D, Brownie S, English M, Gathara D. Informal task-sharing practices in inpatient newborn settings in a low-income setting—A task analysis approach. Nurs Open. 2020;7(3):869-78.

63. City and Guilds. City & Guilds RQF (NVQ) Level 2 Diploma in Health & Social Care online: City & Guilds; 2022 [cited 2023 April 2023]. Course guide]. Available from: <https://www.cityandguilds.com/qualifications-and-apprenticeships/health-and-social-care#fil=uk>.

64. Griffiths P, Ball J, Murrells T, Jones S, Rafferty AM. Registered nurse, healthcare support worker, medical staffing levels and mortality in English hospital trusts: a cross-sectional study. BMJ Open. 2016;6(2):e008751.

65. The North-West Accident and Emergency Managers' Forum. Role of the health care support worker in the A & E department. The North-West Accident and Emergency Managers' Forum. Accid Emerg Nurs. 1997;5(3):131-3.

66. Vaughan S, Melling K, O'Reilly L, Cooper D. Understanding the debate around regulation of support workers. British Journal of Nursing. 2014;23(5):260-3.

67. Walker MJ. Effects of the medication nursing assistant role on nurse job satisfaction and stress in long-term care. Nursing Administration Quarterly. 2008;32(4):296-300.

68. National Health Service. Healthcare support worker <https://www.healthcareers.nhs.uk/explore-roles/healthcare-support-worker/roles-healthcare-support-worker/healthcare-support-worker>: NHS; n.d [cited 2023 February 2023].

69. Nabudere H, Asiimwe D, Mijumbi R. Task shifting in maternal and child health care: An evidence brief for Uganda. International Journal of Technology Assessment in Health Care. 2011;27(2):173-9.

70. World Health Organisation. WHO recommendations: Optimizing health worker roles to improve access to key maternal and newborn health interventions through task shifting. In: WHO, editor.: WHO Optimize MNH; 2012, 2014. p. 98.

71. Arnon Z, Ben-Arye E, Attias S, Levy O, Schiff E. Integrative medicine as a change agent of hospital staff: From hospital orderlies to partners in health promotion. European Journal of Integrative Medicine. 2018;18:42-6.
