# Supplementary material for: Characterising support and care assistants in formal hospital settings: a scoping review
Source: Hum Resour Health. 2023 Nov 27;21:90. doi: 10.1186/s12960-023-00877-7 (PMC10680191; doi:10.1186/s12960-023-00877-7)
Supplement: Supplementary file 7 — Additional file 7. Outcome mapping from sources of evidence. Mapping whether sources report on the outcomes of interest. [file 12960_2023_877_MOESM7_ESM.docx]

# *Additional file 7: Outcome mapping from sources of evidence*

| **Authors, Year** | **Country** | **Region**  **(Income group)** | **Reported or Mentioned (Yes/No)** | | | | | |
| --- | --- | --- | --- | --- | --- | --- | --- | --- |
|  |  |  | **Duties of the Ward Assistants** | **Training** | **Patient care outcomes** | **Patient Experience of care** | **Nurses Experiences and Sentiments** | **Regulatory/ Clinical governance mechanism** |
| Cartwright et al. 2021 | Australia | Western Pacific  (HIC) | Yes | Yes | Yes | No | No | No |
| Duffield et al. 2018 |  |  | Yes | No | Yes | Yes | Yes | No |
| Duffield et al. 2019 |  |  | Yes | Yes | Yes | Yes | Yes | Yes |
| Open Colleges et al. 2022 |  |  | No | Yes | No | No | No | No |
| Roche et al. 2016 |  |  | Yes | No | Yes | No | No | No |
| Roche et al. 2017 |  |  | Yes | Yes | No | No | No | No |
| TAFE SA 2022 |  |  | No | Yes | No | No | No | No |
| Hirose et al. 2022 | Japan |  | Yes | No | Yes | No | No | Yes |
| Chang et al. 1995 | Hong Kong; China |  | Yes | No | No | No | No | No |
| Tou et al. 2020 | Taiwan, China |  | Yes | Yes | No | No | Yes | Yes |
| Tzeng et al. 2004 |  |  | Yes | Yes | No | Yes | Yes | Yes |
| Yang et al. 2015 |  |  | Yes | No | Yes | No | Yes | Yes |
| Peduzzi et al. 2006 | Brazil | South America (UMIC) | Yes | No | No | Yes | Yes | Yes |
| Barken et al. 2015 | Canada | North America  (HIC) | Yes | No | No | No | No | Yes |
| McCloskey et al. 2015 |  |  | Yes | No | No | No | No | No |
| MacKay et al. 2014 |  |  | Yes | Yes | Yes | Yes | No | No |
| Mallidou et al. 2013 |  |  | Yes | No | No | Yes | No | No |
| Zeytinoglu et al. 2014 |  |  | Yes | Yes | No | No | No | Yes |
| Abrahamson et al. 2020 | USA |  | Yes | Yes | No | Yes | No | No |
| American Red Cross 2022 |  |  | No | Yes | No | No | No | Yes |
| Castle et al. 2011 |  |  | No | Yes | Yes | No | No | Yes |
| Franzosa et al. 2018 |  |  | Yes | No | No | No | No | No |
| Gould et al. 1996 |  |  | Yes | Yes | No | No | No | No |
| Handschu et al. 1973 |  |  | Yes | Yes | No | No | No | No |
| Hyer et al. 2011 |  |  | No | No | Yes | No | No | No |
| McMullen et al. 2015 |  |  | Yes | Yes | No | No | No | Yes |
| National Council of State Boards of Nursing 2016 |  |  | Yes | No | No | No | No | Yes |
| Nyberg et al. 1997 |  |  | Yes | Yes | No | No | No | Yes |
| Smith et al. 2001 |  |  | Yes | No | No | No | Yes | No |
| Trinkoff et al. 2017 |  |  | No | Yes | Yes | No | No | Yes |
| Ward et al. 2014 |  |  | No | Yes | No | No | No | No |
| Blay et al. 2020 | Global | NA | Yes | Yes | No | No | No | YES |
| Hewko et al. 2015 | Global | NA | Yes | Yes | No | No | No | Yes |
| Just et al. 2021 | Global | NA | Yes | No | No | Yes | No | No |
| McKenna H. et al. 2004 | Global | NA | Yes | Yes | Yes | No | Yes | Yes |
| Vaughan et al. 2014 | Global | NA | Yes | No | No | No | Yes | Yes |
| Walker et al. 2008 | Global | NA | No | No | No | No | No | No |
| WHO et al. 2008 | Global | NA | No | No | No | No | No | Yes |
| WHO OptimizeMNH 2014 | Global | NA | Yes | No | No | No | No | Yes |
| WHO OptimizeMNH 2012 | Global | NA | Yes | No | No | No | Yes | Yes |
| Arnon et al. 2018 | Israel | European  (HIC) | Yes | Yes | Yes | Yes | Yes | No |
| Hasson et al. 2005 | Republic of Ireland |  | Yes | No | No | No | No | No |
| Furaker et al. 2008 | Sweden |  | Yes | No | No | No | No | No |
| Gransjön Craftman et al. 2016 |  |  | Yes | No | No | No | Yes | Yes |
| Arblaster et al. 2004 | United Kingdom |  | NA | Yes | No | No | No | Yes |
| Bach et al. 2008 |  |  | Yes | Yes | No | No | Yes | No |
| Bosley et al. 2008 |  |  | Yes | Yes | Yes | Yes | Yes | Yes |
| Burns et al. 2007 |  |  | Yes | No | No | Yes | Yes | No |
| City and Guilds et al. 2022 |  |  | No | Yes | No | No | No | No |
| Duffield et al. 2014 |  |  | Yes | Yes | Yes | No | Yes | Yes |
| Faulkner et al. 2016 |  |  | Yes | Yes | Yes | Yes | No | No |
| Francomb et al. 1997 |  |  | Yes | No | No | No | No | No |
| Griffiths et al. 2016 |  |  | Yes | No | Yes | No | No | No |
| Griffiths et al. 2019 |  |  | NA | No | Yes | No | No | No |
| Hancock et al. 2006 |  |  | Yes | Yes | No | No | No | No |
| King et al. 2009 |  |  | Yes | Yes | No | No | Yes | Yes |
| National Health Service 2023 |  |  | Yes | No | No | No | No | No |
| National Health Service 2023 |  |  | Yes | No | No | No | No | No |
| Skills for Health et al. 2020 |  |  | No | Yes | No | No | No | No |
| Spilsbury et al. 2005 |  |  | Yes | Yes | Yes | No | Yes | Yes |
| Spilsbury et al. 2004 |  |  | Yes | No | No | Yes | No | No |
| The Cavendish Review 2013 |  |  | Yes | No | No | No | Yes | Yes |
| The North-West Accident and Emergency Managers' Forum. 1997 |  |  | Yes | Yes | No | No | Yes | Yes |
| Thornley et al. 2000 |  |  | Yes | Yes | No | No | No | Yes |
| Warr et al. 2002 |  |  | Yes | No | No | No | Yes | No |
| Weir et al. 2015 |  |  | Yes | Yes | No | No | Yes | No |
| Wild et al. 2011 |  |  | No | No | No | No | No | No |
| Jennings et al. 2011 | Benin | African Region  (LMIC) | Yes | Yes | Yes | Yes | Yes | No |
| KWTRP et al. 2018 | Kenya |  | Yes | Yes | No | No | No | Yes |
| Ministry of Health 2014 |  |  | No | No | No | No | No | No |
| Omondi et al. 2020 |  |  | Yes | No | No | No | No | No |
| Olson et al. 2013 | Malawi | African Region  (LIC) | Yes | Yes | Yes | No | No | Yes |
| Nabudere et al. 2011 | Uganda |  | Yes | No | No | No | No | Yes |
