# Supplementary material for: Characterising support and care assistants in formal hospital settings: a scoping review
Source: Hum Resour Health. 2023 Nov 27;21:90. doi: 10.1186/s12960-023-00877-7 (PMC10680191; doi:10.1186/s12960-023-00877-7)
Supplement: Supplementary file 8 — Additional file 8. Regulatory and clinical governance mechanisms. List of regulatory and clinical governance mechanisms as reported from individual countries and sources. [file 12960_2023_877_MOESM8_ESM.docx]

# *Additional file 8: Regulatory and clinical governance mechanisms*

| Authors, Year | Country | Nature of legislation/regulatory/governance mechanism |
| --- | --- | --- |
| Duffield et al. 2019,  Blay et al. 2020 | Australia  (HIC) | **Training requirements:**   - Requirement for mandatory completion of Certificate III in Allied Health assistance   **Practice**:   - No formal regulation of Nursing Assistant (NA's) but practice guidelines on tasks a Nursing Assistant could undertake, - Direction and supervision by a qualified registered nurse - Western Australia: Tasks allocation/delegation to be done by a registered and nurse and should be within the Scope of Nursing Practice Decision Making Framework. |
| Peduzzi et al. 2006 | Brazil  (UMIC) | **Training and Practice requirements:**   - Nursing workforce Law 7, 498/86 prohibits work of unlicensed nurses or without formal technical education. |
| Barken et al. 2015,  Zeytinoglu et al. 2014 | Canada  (HIC) | **Training requirements:**   - No institution that oversees PSW ongoing professional development as a requirement for the continuation of their certification   **Practice:**   - No regulation for Patient Support Workers (PSW) - Community Care Access Centres are in charge of performance management contracting - Community Care Access Centres (Ontario) have a client services policy with Ministry of Health and Long-term Care Facilities that mandate home care agencies to develop local guidelines for transferring tasks from health-care professionals to home-care workers. |
| Hirose et al. 2022 | Japan  (HIC) | **Training requirements:**   - The Ministry of Health encourages hospitals to provide in-hospital training more than once a year.   **Practice:**   - Japanese Ministry of Health, Welfare and Labour defines nurse aides and the tasks they should perform. - The ministry has an additional economic incentive for hospitals with a higher nurse aide staffing level per bed. |
| Health Services Unit KEMRI Wellcome Trust, 2018 | Kenya  (LMIC) | **Training requirements:**  None  **Practice**:   - Proposal: A Neonatal HCA to be under the supervision of a registered nurse - To be delegated tasks within a defined scope of practice, and to work in the neonatal unit (NBU). |
| Olson et al. 2013 | Malawi  (LIC) | **Training requirements:**  None  **Practice**:   - Reporting and supervision on the ward by a senior nurse and programme coordinator |
| Gransjön Craftman et al. 2016 | Sweden  (HIC) | **Training requirements:**  None  **Practice:**  The Swedish National Board of Health and Welfare (SNBHW) has issued statutes on the delegation of medical tasks to unlicensed personnel. That is:   - The delegation must not compromise patient safety - It’s an individual arrangement between the RN as delegator and the UAP as the receiver of the delegation is accountable for both accepting the delegation and the actions taken in executing it. - The delegation is time-limited and must be renewed once a year to be valid. - In keeping with the obligation of safe practice, the nurse delegator should decline delegating a task if he or she anticipates that it would not be in the resident’s best interest. |
| Tou et al. 2020,  Tzeng et al. 2004,  Yang et al. 2015, | Taiwan, China  (HIC) | **Training requirements:**   - A Nurse Aide must acquire a certificate (Nursing aide certificate/Technician certificate) before starting work as an Aide. - Taiwan passed a 1993 legislation that regulates pre-job training and the non-specialist status of nurse aides. - The 1993 Act requires that any accredited training program for nurse aides to include 60 hours of lecture and 40 hours of clinical practice. - The content of the training should cover basic nursing, communication, emergent management skills. - Also, teaching hospitals and nursing homes involved in clinical practice training should have bed capacity of at least 50.   **Practice**:   - Tasks of nurse aide are assigned by a registered nurse. - Taiwan government (2005) initiated use of several nursing skill mix models: and assignment of specific duties to nurse aides |
| Nabudere et al. 2011 | Uganda  (LIC) | **Training requirements:**  None  Practice:   - Highlights proposed task shifting Policy for lay health workers. - Phase out of nursing assistants by the Uganda’s Ministry of Health due to reluctance by nurses and clinical officers to delegate tasks and supervise the nursing assistants |
| Arblaster et al. 2004,  Blay et al. 2020,  Bosley et al. 2008,  Duffield et al. 2014,  King et al. 2009,  Spilsbury et al. 2005,  The Cavendish Review 2013,  The North-West Accident and Emergency Managers' Forum. 1997,  Thornley et al. 2000,  McKenna H. et al. 2004,  Vaughan et al. 2014, | United Kingdom  (HIC) | **Training requirements:**   - Healthcare assistants have no compulsory or consistent training, and a profusion of many job titles. In the UK, the nursing regulatory body (UKCC 1986) and the Department of Health (DoH) (DHSS 1987) proposed the title HCA. Although this has not been enforced in any legal sense, HCA is now the most commonly accepted title, and they would be officially recognized as an integral part of the nursing workforce for national negotiations on pay and service. - Apart from NVQs there are no national mandatory educational programmes for HCAs in the UK. However, City and Guilds is the awarding body for NVQ in Care. Progressing from NVQ level 2 to 3 one must meet some criteria (i.e., senior support works, training as a vocational assessor, willing to start nurse training). NVQ competency training centre undergoes both internal and external quality monitoring and audit. City and Guilds has a (A-E) star rating of NVQ accredited centres as part of quality assurance. The NVQ is a form of skill-based classification system. - Beginning 2005, Care assistants could be admitted to the Royal College of Nursing (RCN) as associate members. - RCN (2003) has published a guidance (2003) on indicators for HCA role in critical care. That is, services should only consider developing the HCA role if there is benefit to the patient. Also, HCA role need to be trained and assessed to SVQ/NVQ (Scottish/National Vocational Qualifications) level 2/3. - The Cavendish review proposes a “Certificate of Fundamental Care,” and asks that the Care Quality Commission [CQC] require all workers to have achieved this Certificate before working unsupervised. The Certificate would link healthcare assistant training to nurse training for the first time. However, Most NHS Trusts theoretically require HCAs to have NVQ Level 2 on joining at Band 2   **Practice**:   - Nursing and Midwifery Council (NMC) emphasises the responsibilities of the RN in delegation of patient care to the HCA - need to consider the HCA's knowledge, experience, skills, and competencies [not quite explicit though] - Supervision: Support workers to work under the direction of a qualified nurse: A ratio of 1:1 nurse to HCSW supervision is recommended NVQ accreditation has now provided both the basis for a formal recognition of experiential learning and also the means by which progress might be made either into registered nurse training, or even along parallel - and more practice-orientated lines - However, in practice, HCAs reported and were observed, working predominantly alone, providing bedside care to patients without the support and supervision of registered nurses. - HCAs are not currently subject to professional regulation and as a result are not professionally accountable. - HCAs work in ways that are not always reflected in the formal arena of official policies. - The Agenda for Change showed the clear intention to allow HCAs to take on more tasks currently carried out by nurses. In response, attempts have been made to develop skills, experience, and career ladders for HCAs. As part of the modernization of the NHS the DoH (2000a) have introduced the skills escalator to encourage all staff, including HCAs, to acquire additional skills and develop their roles and expand their experience. - In 2004, the Royal College of Nursing (RCN) incorporated the HCA within the nursing family echoing the Royal College of Midwives (1999) who welcomed HCAs as members of the maternity team. - Health service providers in UK have a number of legal duties with regard to employing support workers. - NHS employers must meet agreed employment check standards, including checking qualifications, employment history, references, criminal record and, with regard to registered staff, registration status (NHS Employers, 2013). - In Scotland and Wales, codes of conduct have been developed for HCSWs (The Scottish Government, 2009a; NHS Wales, 2011a), which ‘mirrors what is required of all ‘regulated’ healthcare professionals [they] may work alongside’. - Skills for Health(organisation) has also developed core standards specifically for assistant practitioners (Skills for Health, 2009). Separate codes of practice for employers call for them to implement systems and processes to support HCSWs to achieve the standards in the codes of conduct and provide workplaces that promote development and fulfilment (NHS Wales, 2011b; The Scottish Government, 2009b). - The Cavendish Review (2013) into HCSWs in the NHS and social care settings proposed the development of standards with regard to the employment and training of HCSWs; Correct processes of delegation and accountability; legal liabilities to act in the best interest of the patient; employment law; codes of conduct; codes of practice and the ongoing development of core training standards. Also, proposed health and social care professionals who work in the UK must be registered with one of 12 regulators. More standardised processes of recruitment, selection, and interview; a common set of standards for practice; an agreed code of conduct for support workers across the UK and minimum training qualifications before worker |
| American Red Cross 2022,  Castle et al. 2011,  McMullen et al. 2015,  National Council of State Boards of Nursing 2016,  Nyberg et al. 1997,  Trinkoff et al. 2017,  Blay et al. 2020,  Duffield et al. 2019 | USA  (HIC) | **Training**:   - Any nurse assistant training program should deliver an engaging curriculum that meets state and federal education regulations. - Federal regulations concerning CNA training and competency, were established by the Omnibus Budget Reconciliation Act of 1987 - These regulations require nursing homes to employ CNAs who complete state-approved CNA programs, outline the fundamental skills that should be included in all CNA programs, and require the CNA to pass a competency evaluation administered and evaluated only by the state or by a state-approved entity and be added to the state registry. - Each state to main a registry of NAs who have met state and federal training requirements. - The regulations define the minimum curriculum to be included in a CNA program but do not necessarily define all the activities, skills, or procedures that can be performed by a CNA. - Development of a competency-based orientation program that helps UAP develop and maintain their specific skills. - Code of Federal regulations 2012 require at least initial training hours of 75(at least 16 clinical hours and 12 in-service training hours). About 60% of US states have requirement for additional training hours for CAN working in hospitals or nursing homes.   **Practice**:   - State defined regulations/guidelines for RN delegation, supervision of unlicensed assistive personnel (support workers), scope of practice varies from one state to another. - The Centres for Medicare and Medicaid Services (CMS) requires nursing homes to employ certified nursing assistants or aides (CNAs) as part of a mechanism to ensure higher standards of care. - CMS defers to state requirements for what CNAs are allowed to perform. - Federal government regulations are not specific to Nurse aides; but require that a facility provides services by a sufficient number of nursing personnel on a 24-hour basis to provide required care in accordance with care plans. - Code of Federal Regulations - 42 CFR, 483: lists some CNA scope of practice, though not official. Focuses on care for residents in nursing facilities. The CFR guides training and duties for CNAs. - Scope of delegated tasks may vary from one state to another. - The US National Council of State Boards of Nursing enumerates the five rights of delegation - right task, circumstances, person, direction, supervision. |
| WHO 2008,  WHO OptimizeMNH 2012, 2014 | Global | **Practice**:   - The WHO global recommendations and guidelines on task shifting in the context of HIV/AIDS care. - The guidelines could be adopted as national strategy for organising and optimising the health workforce in HIV/AIDS programme |
|  |  | - Provides recommendations for optimizing task shifting roles that can be safely and effectively be delivered by lay health workers particularly auxiliary nurse and auxiliary nurse midwife in maternal and newborn care programmes. |
