# Supplementary material for: Characterising support and care assistants in formal hospital settings: a scoping review
Source: Hum Resour Health. 2023 Nov 27;21:90. doi: 10.1186/s12960-023-00877-7 (PMC10680191; doi:10.1186/s12960-023-00877-7)
Supplement: Supplementary file 9 — Additional file 9. Topics/skills covered during Care Assistants’ training. List of training topics/content. [file 12960_2023_877_MOESM9_ESM.docx]

# *Additional file 9: Topics/Skills covered during ward/care assistants training*

| Training topic | Frequency of mention |
| --- | --- |
| Basic Nursing Care | 42 |
| Vital Signs taking |  |
| Specimen Collection |  |
| Anthropometric Measurements |  |
| Simple Wound Care |  |
| Workplace Health and Safety | 17 |
| Communication Skills | 16 |
| Other Soft Skills | 11 |
| Infection Prevention and Control | 8 |
| Equipment Processing and Preparations |  |
| First Aid and Emergency Preparedness | 8 |
| Anatomy and Physiology (body systems) | 7 |
| Confidentiality, Privacy and Dignity | 7 |
| Record Keeping and Documentation | 7 |
| Medical Terminology | 5 |
| Handling Diverse and Difficult People | 5 |
| Palliative Care Support | 5 |
| Clerical Skills | 4 |
| Patient Transport | 3 |
| Basic Counselling | 3 |
| Human Growth and Development | 3 |
| Food and Nutrition | 3 |
| Health Promotion Messaging | 3 |
| Family Support and Centred Care | 1 |
